# Bio-imaging with the helium-ion microscope: A review

**DOI:** 10.3762/bjnano.12.1

**Published:** 2021-01-04

**Authors:** Matthias Schmidt, James M Byrne, Ilari J Maasilta

**Affiliations:** 1 Helmholtz-Centre for Environmental Research GmbH - UFZ, Permoserstraße 15, 04318 Leipzig, Germany; 2 School of Earth Sciences, University of Bristol, Wills Memorial Building, Queens Road, Bristol BS8 1RJ, United Kingdom; 3 Nanoscience Center, Department of Physics, University of Jyväskylä, P.O. Box 35, FI-40014 Jyväskylä, Finland

**Keywords:** bio-imaging, flood gun, helium-ion microscopy, high resolution, HIM, HIM-SIMS, ionofluorescense

## Abstract

Scanning helium-ion microscopy (HIM) is an imaging technique with sub-nanometre resolution and is a powerful tool to resolve some of the tiniest structures in biology. In many aspects, the HIM resembles a field-emission scanning electron microscope (FE-SEM), but the use of helium ions rather than electrons provides several advantages, including higher surface sensitivity, larger depth of field, and a straightforward charge-compensating electron flood gun, which enables imaging of non-conductive samples, rendering HIM a promising high-resolution imaging technique for biological samples. Starting with studies focused on medical research, the last decade has seen some particularly spectacular high-resolution images in studies focused on plants, microbiology, virology, and geomicrobiology. However, HIM is not just an imaging technique. The ability to use the instrument for milling biological objects as small as viruses offers unique opportunities which are not possible with more conventional focused ion beams, such as gallium. Several pioneering technical developments, such as methods to couple secondary ion mass spectrometry (SIMS) or ionoluminescence with the HIM, also offer the possibility for new and exciting research on biological materials. In this review, we present a comprehensive overview of almost all currently published literature which has demonstrated the application of HIM for imaging of biological specimens. We also discuss some technical features of this unique type of instrument and highlight some of the new advances which will likely become more widely used in the years to come.

## Review

### Introduction

**Figure 1:**
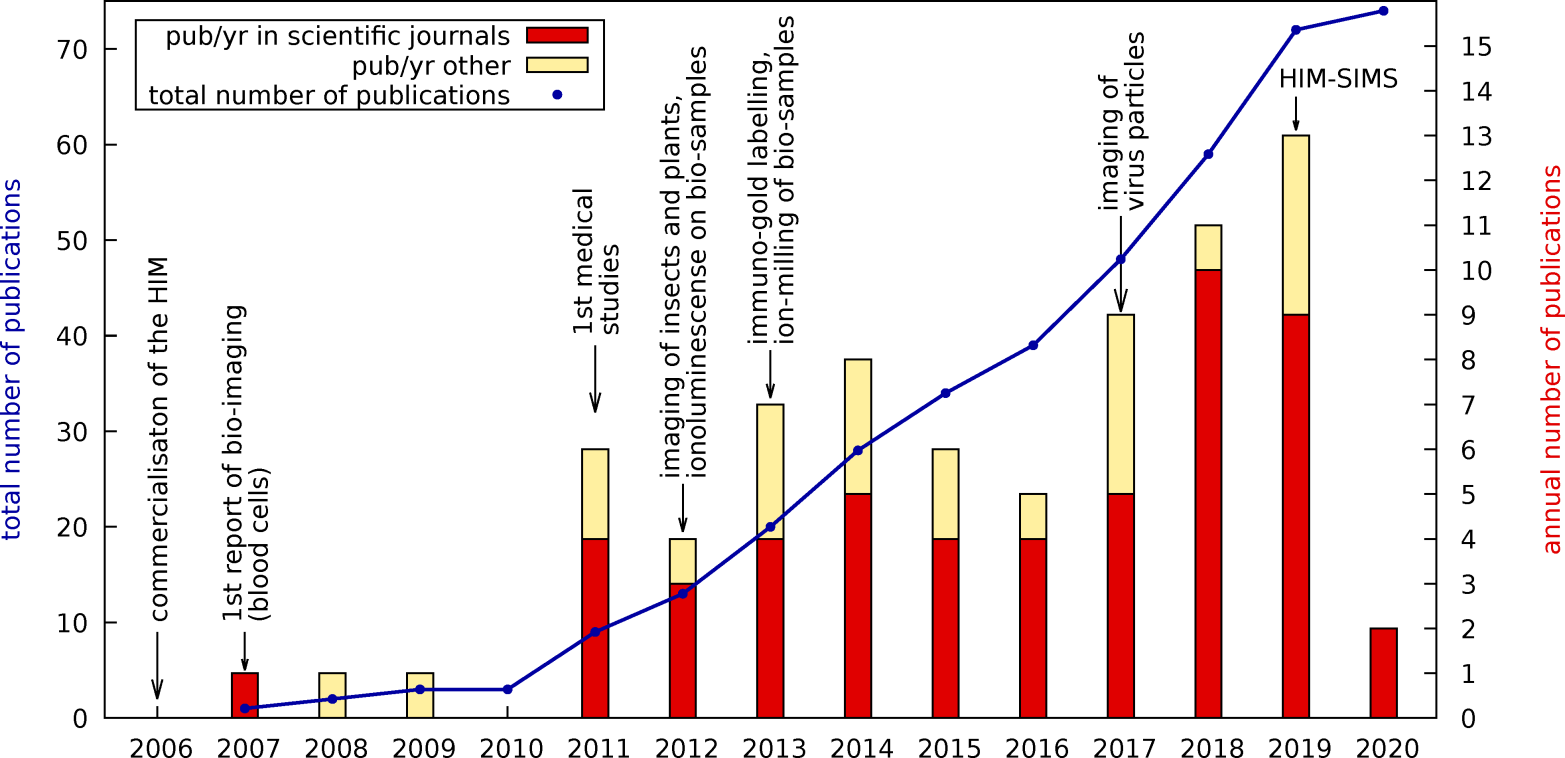
Annual (bars) and accumulated (blue line) numbers of publications on bio-imaging using the helium-ion microscope since its commercialisation. Some important milestones are also indicated. Red bars refer to annual publications in peer-reviewed scientific journals, yellow bars list other publications, such as white papers, Ph.D. theses, and extended conference abstracts.

Since its commercialisation in 2006 [1–5], the helium-ion microscope (HIM) has become a well-established tool for nanoscale imaging and nanoscale fabrication in physics and materials science. It is attractive for those applications as it combines high-resolution imaging of insulating samples with nanoscale milling capabilities in one instrument. The milling efficiency can also be increased by the use of heavier ion species, such as Ne or Ga, where Ne is available for the standard He column, whereas Ga requires an additional column. In contrast to its success in materials science, HIM is much less frequently used for imaging biological specimens. To date, 13 years after the first HIMs were commissioned, only about 70 papers (we are aware of 72) have been published which include HIM bio-imaging data for medical, geomicrobiological, or life sciences. Figure 1 provides an overview of some key applications of HIM in bio-imaging together with an indication for the growth in the volume of literature which has been published in related fields. This steady growth of publications gives an indication for the increasing demand for HIM in biological applications and the opportunity for further developments. On the one hand, it is as flexible and straightforward to use as a scanning electron microscope (SEM) but with a five times larger depth of field [1] and a lateral resolution of about 0.5 nm [4] (demonstrated record: 0.24 nm [5]), which is between high-end field-emission SEMs (FE-SEM) and transmission electron microscopes (TEM). On the other hand, HIM is less demanding in terms of sample preparation compared to both SEM and TEM. In particular, the advantage of HIM is that opaque and non-conductive specimens, which possess a relatively strong topography, can be imaged. This is possible owing to the combination of a large depth of focus and the possibility of charge compensation [6], by pointing an electron beam emitted from a flood gun, onto the area of analysis.

The first HIM micrographs of biological specimens were published between 2007 and 2010 [2,5,7], but did not immediately trigger a wave of follow-up studies. At that time, most of the typical applications for SEMs did not demand a “better” SEM. If they did, the issues appeared to be solvable by the emerging technique of ultralow-voltage SEM [8]. Furthermore, HIM was not yet ready, and, in fact, is still not ready, for studying the finest details of the ultrastructure of cells or to resolve protein structures, which is state of the art in modern transmission electron microscopy. Another obstacle which had to be overcome was the absence of in situ chemical nanoscale analytical tools for the HIM, which were unavailable due to a lack of X-rays created under a 30 kV He^+^ beam due to conservation of momentum. This is in stark contrast to SEM in which X-ray detection methods, such as energy dispersive X-ray spectoscopy (EDX), are more or less available as standard.

The turning point for bio-imaging with HIM in the field of medicine came in 2011 when Bazou et al. used the HIM to study tumor cells [9–10] and Arey et al. studied the interaction of nanoparticles with alveolar epithelial cells [11]. In the following two years, reports on HIM imaging of the nanostructures on butterfly scales by Boden et al. [12], fruit flies by Boseman et al. [13], and pine leaves by Kim [14] marked the entrance of HIM into general biology. Soon afterwards, two more milestones were reached. Firstly, Rice et al. [15] successfully imaged 15 nm gold labels on cell-surface proteins in rat kidneys, which demonstrated that HIM is compatible with the powerful technique of immunogold labelling. Secondly, Joens et al. introduced the ion-beam milling capabilities of the HIM to biological applications when they opened up the mouth cavity of a nematode [6]. In the same paper, Joens et al. furthermore demonstrated on mitotic HeLa cells that HIM is ready for ultrastructure research in cell biology. Research on the ultrastructure of cells with the HIM was subsequently continued by Schürmann et al., who presented HIM micrographs of cell-membrane nanodomains in mammalian cells [16].

HIM entered the field of virology in 2017 when Leppänen et al. used the technique to image T4 bacteriophages infecting *Escherichia coli* bacteria for the first time [17]. Images of a phage-infected bacterium in a sample from the environment was then presented by Sharma et al. in the following year [18]. The possibility to image the attachment of bacterial parasites to the outer membranes of bacteria at high resolution in, at least compared to TEM, a relatively natural state makes HIM a very powerful tool for the investigation of living antibiotics. The work on the life cycle of the bacterial predator *Bdellovibrio bacteriovorus* by Said et al. [19] and a study on the pH-responsive encapsulation of bacteriophages for phage therapy by Vinner et al. [20] are first examples.

Since the early days of HIM, attempts have been made to add nanoscale analytics to the HIM. Already in 2007, Notte et al. stated in their article “An Introduction to the Helium Ion Microscope” [3]: “We have observed that there is photon production from certain materials as the helium beam enters the sample. As with the standard cathodoluminescense effect, we expect that these photons may reveal information about the materials.” With regard to the potential of using the HIM for secondary ion mass spectroscopy (SIMS) they wrote: “[If] a heavier gas in addition to or in place of helium [would be used] the resulting beam [c]ould then be used to generate enough secondary ions to permit SIMS analysis.” Both ideas were quickly proven to be correct in the following years. Ionoluminescense in the HIM (IL-HIM) was used by Veligura et al. to investigate NaCl and semiconductor materials [21–23]. Franklin first investigated the suitability of IL-HIM for studying biological specimens tagged with fluorescent markers [24]. Another application in bio-imaging was published in 2018 by Sato et al., who used the ionoluminescense generated by the He ion beam to detect ZnO nanoparticles which were incubated with COS7 cells [25]. Today HIM-SIMS is possible via two different approaches. The first, a sector-field mass-spectrometer SIMS, was developed by Dowsett, Wirtz, et al. [26–29], and commercialised by Carl Zeiss Microscopy [30]. The second approach, developed by Klingner, Hlawacek et al., integrated a spectrometer for Rutherford backscattering analysis with time-of-flight (ToF) SIMS for the HIM [31–35]. The first biological application of HIM-SIMS was published by Lovric et al., who investigated *E.coli* bacteria exposed to TiO_2_ nanoparticles using the sector-field SIMS spectrometer [36]. In this review article, we build upon previous articles by Kim [37] and Gölzhäuser and Hlawacek [38] to present an overview on past discoveries and recent developments reported for bio-imaging using HIM for biological, medical, plant, animal, microbiology, virology, and geomicrobiology studies. We briefly discuss the imaging, detection, and analytical technologies which make the HIM so powerful and explain why these technologies have been so beneficial to biological applications. We have highlighted, to the best of our knowledge, most publications which include HIM bio-imaging and have separated these articles into specific categories to provide the reader with a quick and concise overview of their particular field, or fields, of interest. We also briefly touch upon some of the most commonly applied preparation methods which are applicable to both electron and ion microscopy. Overall, this review has been written to inspire new and exciting studies using this powerful imaging technique based on helium ions. We expect that the next decade will see some remarkable discoveries, especially as the combination of high-resolution imaging with nanoscale analytics becomes more widespread.

### Resolution and contrast mechanisms in the HIM

**Figure 2:**
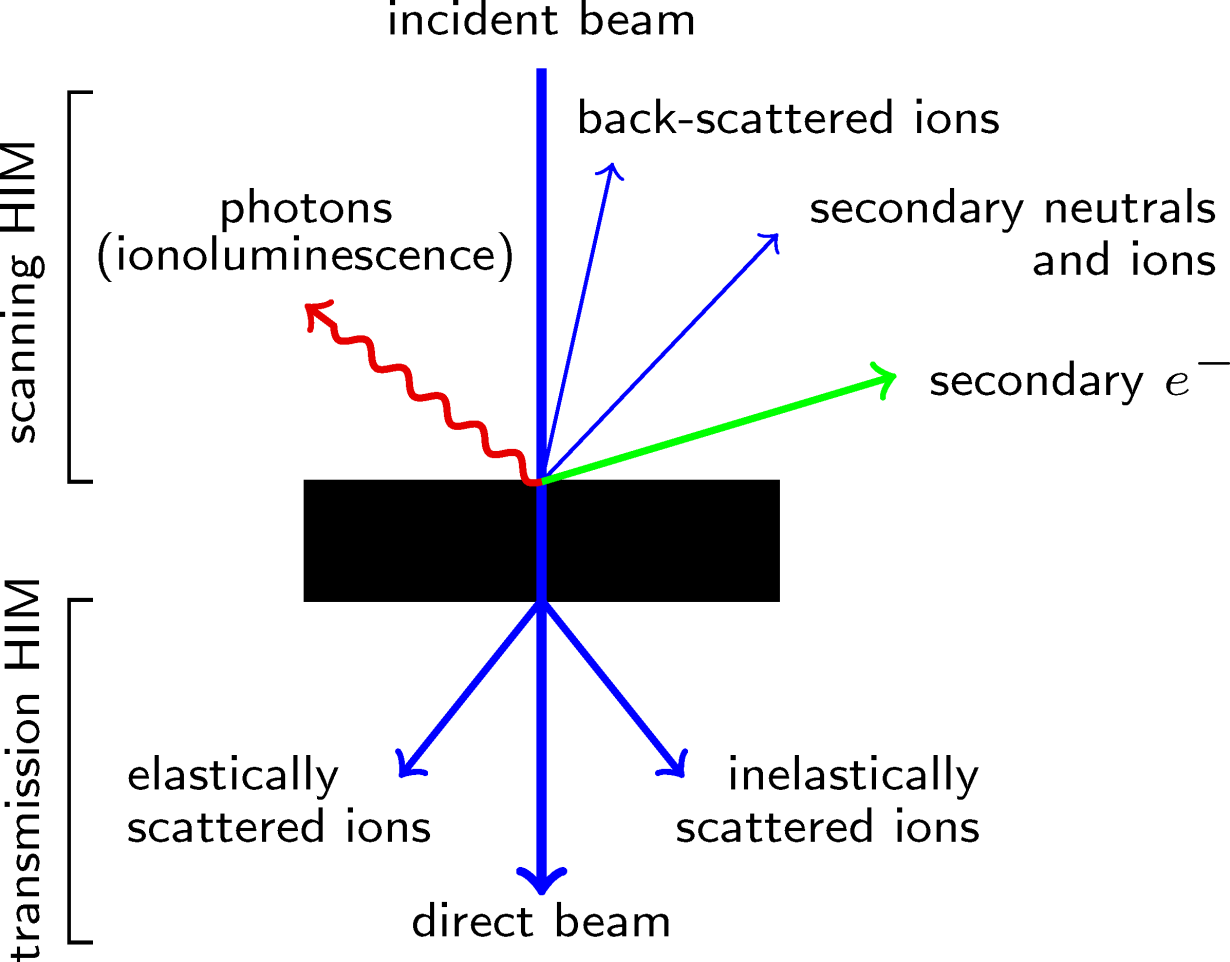
When the ion beam of the HIM interacts with the sample, primary ions and secondary particles escape the sample and can be detected. The figure schematically depicts particles which are, in principle, detectable in the scanning HIM and, in the case of a sufficiently thin sample, in the transmission HIM.

The key to high-resolution imaging with a scanning microscope is a high-brilliance source of small size. In the HIM this is realised by the atomic level (or gas field) ion source, which, in essence, is a single tungsten atom at which the gas atoms are ionised [1,39]. The column optics projects an image of that atom onto the sample, which commonly is referred to as “beam spot”. The achievable lateral resolution in the HIM is naturally determined by the size of the beam spot, which has a minimum threshold of 0.25 nm [1]. In reality, achieving such a small spot size is extremely challenging and is affected by the ion landing energy as well as instrument parameters, such as the choice of aperture and source de-magnification, which both affect the quality of focus and beam shape [40]. However, the lateral and depth resolution of the measurement also depends on the type of detector which is used for the analysis. This can be understood from the interactions of the impinging ions with matter in the sample (Figure 2). Ion collisions with a nucleus in the sample result in (back-)scattering of the primary ion, displacement of atoms in the sample, sputtering of material and generation of phonons (heat). However, incoming ions also undergo many interactions with electrons in the sample, leading to the generation of secondary electrons, photons, and heat.

With the exception of heat generation, every possible ion–solid interaction can, in principle, be used for imaging or nanoscale analysis in the HIM given a suitable detector. We provide a brief overview of the main detectors which are applicable for HIM, though most of them are not commonly available at the moment.

#### Secondary electron imaging

The high secondary electron yield in a HIM, which is significantly higher than that for low-energy electrons in an SEM [41], makes the detection of secondary electrons favourable for imaging. To date, the majority of HIM studies have employed secondary electron imaging using an Everhart–Thornley (ET)-type electron detector [5,42–43]. This is mainly because the ET detector is the standard detector for the HIM, but also because secondary electron imaging provides the highest resolution currently available with a demonstrated lateral resolution of 0.24 nm [5].

Such a high resolution is possible because of the relatively small amount of energy which is transferred from the helium ion to the secondary electron. Employing the formula for a central elastic collision, a 25 keV helium ion transfers as little as 13.7 eV to the secondary electron. In SEM, the acceleration voltage of the electron beam needs to be lowered to yield low-energy secondary electrons. This increases surface sensitivity since only secondary electrons produced directly under the surface will be able to overcome the work function of the sample and reach the detector. In HIM, the emitted secondary electrons already have low energy, which results in a strong edge and topography contrast. Furthermore, the low energies of the secondary electrons in a HIM produce excellent contrast due to changes in the work functions of the materials. An interesting contrast mechanism occurs when HIM is used to study insulating or poorly conducting materials such as most biological specimens. Here, differences in local conductivity result in the accumulation of positive charges under the ion beam, which hampers the emission of secondary electrons and results in a blackening of the charged areas in the micrograph.

#### Charge compensation

**Figure 3:**
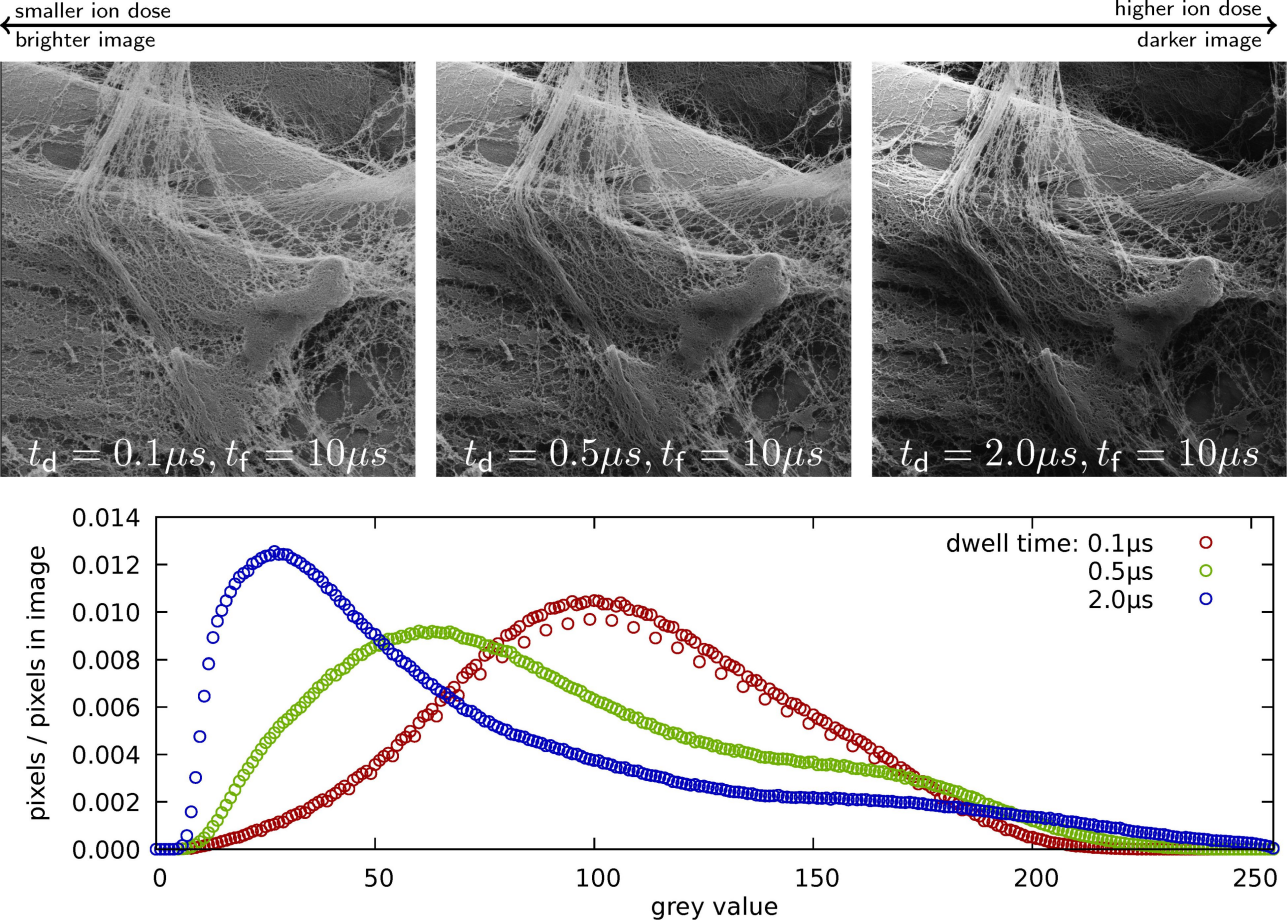
Charge compensation in the HIM using the flood gun. An uncoated maize root was imaged with different dwell times, *t*_d_ = {0.1,0.5,20} μs, while flood time, *t*_f_ = 10 μs, image size, 2048 × 2048 pixels, and brightness/contrast settings of the Everhart–Thornley detector were kept constant. The histograms clearly show a shift towards lower pixel intensities with increasing dwell time. No flooding at all (not shown here) resulted in a black image. The field of view is 20 μm. Unpublished data, sample provided by Yalda Davoudpour.

One of the most frequently cited advantages of using HIM compared to SEM for bio-imaging is the possibility to image typically non-conductive biological specimens without the prior coating with a thin layer of metal to make their surfaces conductive. This is possible due to the development of the charge-compensating electron flood gun, which is one of the flagship features of HIMs [5,43]. The technique is straightforward to use: After having scanned one line, the ion beam is blanked and electrons from the flood gun are directed into the field of view. Then, a new line is scanned followed by another electron flooding. This is particularly useful for biological specimens, which are typically insulators or poor conductors, as it enables imaging at high resolution without depositing a thin conductive layer (e.g., Au, Pt, or C) onto the surface in order to avoid charging. Charge compensation can even be considered to be necessary for conductive metals, because the high surface sensitivity of the HIM would only reveal the metal layer and not the fine detail of the surface ultrastructure without the use of the flood gun. From a practical perspective, charge compensation can be challenging as the total amount of charges required for compensation depends linearly on the number of charges implanted per line scanned. In other words, the settings of the flood gun have to be adjusted whenever the number of pixels per line, dwell time, or beam current are changed. Figure 3 illustrates how the variation of the dwell time of the ion beam on a pixel influences the brightness of the image if the flooding parameters are kept constant. Similar results can be obtained when the flood time is varied at a constant dwell time.

#### Secondary ion mass spectrometry (SIMS)

A major disadvantage of using standard HIM rather than SEM is the lack of analytical detectors for elemental quantification, such as EDX. This is because 30 keV helium ions cannot transfer enough energy to the bound inner-shell electrons of the sample to excite them out of the core states and to enable fluorescent X-ray de-excitation (this would require energies of several kiloelectronvolts). However, recent analytical developments of the HIM have focused on the detection of secondary ions which are sputtered when the primary ion beam of the HIM is scanned across the sample material. The ionised fraction of this material can be extracted by electrostatic extractor optics and passed through a mass spectrometer in order to obtain chemical and isotopic information about the sample. At present, there are two different types of HIM-SIMS available. On the one hand, there is a sector-field SIMS developed by Dowsett, Wirtz et al. [26–30], in which up to four ions can be analysed simultaneously. On the other hand, there is a time-of-flight spectrometer developed by Klingner, Hlawacek et al. [31,33,35]. Both spectrometers have a mass resolution of about 400. So far, HIM-SIMS remains in the development stage with few commercial systems available. However, they are potential game changers for the investigation of biological samples at high spatial resolution in combination with chemical characterisation. A first biological application was presented by Lovric et al. when they investigated *E.coli* which were exposed to TiO_2_ nanoparticles with the sector-field spectrometer [36]. The authors report on secondary ion maps of CN^−^ from the biomass as well as of Ti^+^ from the nanoparticles. So far, the mass resolution achieved does not allow for using HIM-SIMS to characterise isotopically labelled samples, which requires a ten to twenty times higher resolution. To illustrate this, consider a ^13^C-labelled sample. In order to distinguish, for instance, the ion ^12^CH^+^ of mass 13.007 u from ^13^C^+^ of mass 13.003 u a mass resolution of more than about 4000 is needed. If in future HIM-SIMS systems the mass resolutions were improved by about one order of magnitude, HIM-SIMS could enable the localisation of specific bacteria which have metabolised labelled substrates within a microbial community. This type of application is already possible using NanoSIMS instruments. However, such instruments lack the spatial resolving power of the HIM [44]. An interesting and well-characterised geomicrobiological test system for HIM-SIMS spectrometers would be magnetosomes synthesised by magnetotactic bacteria, which are Fe_3_O_4_ nanocrystals of about 50 nm length [45]. HIM-SIMS also appears to be a promising tool to study intracellular deposits of certain chemical elements. An example are phosphate granules in algal biofilms, which were previously investigated using multiple microscopes in a correlative study [46]. Another, certainly more challenging, example would be the identification of iron–sulfur clusters in microbes [47] or mitochondria [48] by HIM-SIMS. Nevertheless, the current mass resolution of the spectrometers allows for several interesting applications in biology, which comprise biomineralisation, microbial corrosion, and interactions between nanoparticles and cells or tissues.

#### Alternative detectors

Rutherford back-scattering (RBS) of high-energy (typically in the range of 1 to 5 MeV) ions is a well-known (micro)analytical technique for the investigation of the elemental composition of a sample. Recently, Klingner, Heller, and Hlawacek demonstrated a time-of-flight RBS spectrometer for the HIM [32,34,49]. We are not aware of any currently published biological application of RBS in the HIM. However, from scattering kinetics it follows that RBS is most sensitive to heavy elements in a light matrix. For bio-imaging this implies that RBS could be a promising technique to study systems containing organic and inorganic matter, for example, the interactions of nanoparticles and cells, immunogold-labelled cells or tissues, and biomineralisation processes, such as the formation of bones or tooth enamel.

In SEM, cathodoluminescence refers to the emission of photons of characteristic wavelengths from a material under electron bombardment. Ionoluminescence (IL) describes the equivalent phenomenon in HIM when free electrons in the sample are excited by the bombardment of the sample by helium ions and emit photons in the range from near-IR to near-UV [3,21–23]. IL spectra as well as the temporal decay of IL after excitation contain information about the emitting material. IL emitted under megaelectronvolt proton or alpha-particle irradiation has proven to be a powerful tool for the investigation of medical and biological samples [50]. In the context of bio-imaging with the HIM, IL-HIM holds promise to detect fluorescent biomarkers with better resolution than that achievable even with the most advanced super-resolution optical microscopy techniques, for instance, stimulated emission depletion microscopy [51]. In particular, it may allow for correlating fluorescence microscopy with HIM. Few bio-imaging studies have focused on the detection of IL. To the best of our knowledge, the only work on IL-HIM bio-imaging was done by Franklin and is published in his Ph.D. thesis [24]. He investigated the IL of fluorescent dyes and applied this to study an Alexa Fluor 488-tagged mouse incisor. However, he found that “the area of interest was becoming increasingly bleached under the beam. It was discovered that only one [IL-HIM] image was achievable before bleaching the sample at a 20 μm field of view.” In order to overcome the ionobleaching of biomarkers, Franklin also tested nanoscale diamonds doped with the fluorescent nitrogen-vacancy defect as well as rare earth metal-based nanoparticles regarding bleaching under the ion beam. Although they are believed to be photostable under electron irradiation, the IL of the nanoscale diamonds decreased significantly under the ion beam even at doses lower than 10^14^ ions·cm^−2^. However, lanthanide-doped nanoparticles proved to be relatively stable against the ion irradiation and seem to be promising materials for biomarkers to be used with the HIM. Later, Mi developed a particle-accelerator-based setup with high-energy protons and alpha particles to excite IL in biological specimens [52]. In this Ph.D. thesis, Alexa Fluor 514-labelled HeLa cells were imaged with 2 MeV protons. Furthermore, optimisation protocols for the design of luminescent lanthanide-doped nanoparticles with a quantum yield of up to 0.673 were presented. In the aforementioned work by Sato et al., HIM imaging was used to study COS7 kidney fibroblast cells [25]. In one of the experiments described in the paper, the cells were incubated with ZnO nanoparticles whose fluorescense was detected by IL-HIM. In general, it can be concluded that IL-HIM is particularly promising for biological applications if ionobleaching of fluorescent bio-markers can be overcome. The few studies available so far suggest to develop bio-markers on the basis of rare earth metal-doped nanoparticles.

The portfolio of possible contrast mechanisms in HIM is not complete without the detection of transmitted ions. Whilst accelerator-based transmission ion-microscopy using protons or alpha particles has been used to investigate biological specimens since the 1980s [53], none of the few (scanning) transmission helium-ion microscopy (THIM) studies using 10 to 40 kV helium ions have imaged biological specimens [5,54–56]. It can be speculated that this is mainly due to a lack of a detector for transmitted ions in the latest-generation HIM (Zeiss Orion NanoFab) [43], although it was a standard detector in the first-generation HIM (Zeiss Orion Plus) [5]. However, THIM is desirable as it would extend the range of applications to thin sections, similar to the transmission option in SEMs. In combination with the well-established heavy-metal staining techniques used in transmission electron microscopy (TEM), this would allow for ultrastructural research comparable to standard TEM. SRIM [57] simulations show that up to 400 nm thick sections of epoxy resins could be penetrated by 30 kV helium ions (unpublished work by M. Schmidt). Even more importantly, THIM is highly complementary to secondary electron imaging in the HIM. It provides insight into an object whereas the latter only images its surface. If a segmented detector with a central segment and ring segments was used, it would be even possible to distinguish between absorption contrast (bright-field imaging) and scatter-contrast (dark-field imaging). Compared to other imaging modes in the HIM, it can be anticipated that THIM will be the best choice for imaging cell organelles, bacterial nanoparticles, or bacteriophages inside infected bacterial cells.

This section would be incomplete without mentioning two interesting new developments. Firstly, Mouseley et al. have developed a full-field THIM, which is not based on a Zeiss Orion instrument, and which holds promise to conduct exciting THIM experiments also on biological samples [58]. Secondly, within the npSCOPE H2020 project an “instrument [is being developed] that couples the extraordinarily high resolution of the […] helium-ion microscope with sensors for composition (a mass spectrometer) and 3D visualization (transmitted ion detector) in order to more fully characterise individual nanoparticles and their interaction with their environment (tissue, cells, etc.) […]” [59].

### Sample preparation

Sample preparation is critical to the success of imaging any biological material at high resolution using electron and ion microscopy techniques. Depending on the sample under investigation, biological samples prepared for HIM might include structures such as bacterial cells, biofilms, exopolymeric substances (EPS), or minerals. The high vacuum applied in HIM means that any liquid remaining in a sample is subject to surface tension, which can lead to damage of the specimen during imaging. To overcome this issue, researchers typically focus on removing any liquid water from a sample whilst, at the same time, maintaining the cellular integrity. This is usually achieved by chemical fixation, followed by dehydration and then drying. For non-conductive samples, as is typical of biological materials, established protocols for SEM also involve methods to overcome charging effects, which are, however, not absolutely necessary for HIM owing to the charge compensation.

#### Fixation

Fixation is often applied as the first step during sample preparation to prevent or limit alterations to biological materials during sample drying. Chemical fixatives such as glutaraldehyde, formaldehyde, or a combination of the two (Karnovsky’s solution) cross-link proteins and lipids to physically stabilise samples [60]. Glutaraldehyde (2–2.5%) fixation is usually performed at cold temperature (4 °C) to avoid the formation of artifacts and is well suited for samples containing a high density of cells, such as biofilms. The length of the fixation time should be varied depending on the sample. Before fixation, specimens are also sometimes treated with additional chemicals, such as 1% tannic acid (TA), and/or osmium-fixed to promote membrane integrity [61]. After fixation, water is removed from samples via dehydration in ethanol or methanol with increasing concentrations, for example, 30%, 75%, 95%, and 100% for 10 min each [60,62]. Samples can be stored in about 70% ethanol prior to drying.

#### Drying

The next stage in sample preparation of biological samples focuses on drying to avoid damages to fragile cell surfaces, internal structures, EPS, biofilms, or mineral associations. In the following, a brief overview over different routinely applied drying methods is given. (I) Freeze drying: Samples are frozen (e.g., using a plunge or high-pressure freezer) and then dried under vacuum. Uryu et al. suggested a pathway which includes plunge freezing for instantaneous immobilization followed by freeze drying in a cold nitrogen gas and finally critical point drying [63]. This method (termed FDGN_2_) resulted in unprecedented structural detail during imaging with SEM but yielded various artifacts during imaging with HIM [64]. (II) Critical point drying (CPD): The basic principle of CPD is to substitute ethanol in the dehydration preparation stage with a supercritical fluid, such as carbon dioxide (CO_2_). The temperature and pressure around the sample are raised to the critical point, 31 °C and 7.4 MPa (1073 psi), at which point CO_2_ becomes supercritical. The pressure is then lowered at constant temperature returning the CO_2_ to a gaseous state. Through this process the sample is dried without ever crossing the liquid–gaseous boundary and, thus, avoiding surface tension, which is so damaging to the sample. The operating procedures for CPD vary depending on the sample or instrument, with some being automated and others manually operated. Rice et al. used methanol during the dehydration stage, purged the samples with cold liquid CO_2_, and raised temperature and pressure to, respectively, 42 °C and 1200 psi for equilibration for more than 4 min [15]. The pressure was then reduced (*<*100 psi/min) at constant temperature (32 °C) until the samples were dried. The samples were stored under desiccant at room temperature, and the authors noted no obvious changes to the samples after one week of storage. This protocol was repeated by Paunescu et al. to preserve the morphology of rat and mouse epididymal tissue [65]. (III) Hexamethyldisilazane (HMDS): Low-tension media, such as HMDS, can be added after the final stage of ethanol dehydration. This method is most often used as an alternative to CPD due to the ease and rapidity of application. However, its carcinogenic properties mean that careful handling is necessary. Several studies have applied HMDS treatment for HIM [17,60,64,66]. (IV) Air drying: During air drying, the liquid–gaseous boundary is crossed, leading to strong surface tension, which acts on the sample and causes damage to the structure, leading to agglomeration and collapse. Consequently, air drying should only be used as a last resort [60]. (V) Resin embedding: Samples can be embedded within a resin, such as epoxy, which infiltrates biological material and is later polymerised without affecting the cellular structure. For example, Bidlack et al. investigated tooth enamel, which contains both mineral and organic phases, in a three-dimensional configuration [67]. They found the best preparation method was to first fix the sample chemically and then perform a gradual ethanol substitution with LR White acrylic resin. Samples were then polymerized at 60 °C for 24 h and allowed to cool. The blocks were then polished at room temperature to expose the area of interest within the tooth enamel.

As biological samples are electrical insulators, charging effects typically affect the imaging quality in conventional SEM. Various approaches are used to remove the charge, including coating with electrically conductive materials, such as carbon, platinum, or gold. Coating the samples can, however, result in minute changes to surface features which are only visible at very high resolution. One of the main advantages of HIM is the ability to neutralise charge by implementation of an electron flood gun. This flood gun eliminates the need for coating biological samples with conductive materials to obtain high-resolution information. In fact, this is often cited as one of the main benefits of using HIM and has been reported in a number of publications focusing on biological samples [11,62,65].

#### Ionic liquid preparation

**Figure 4:**
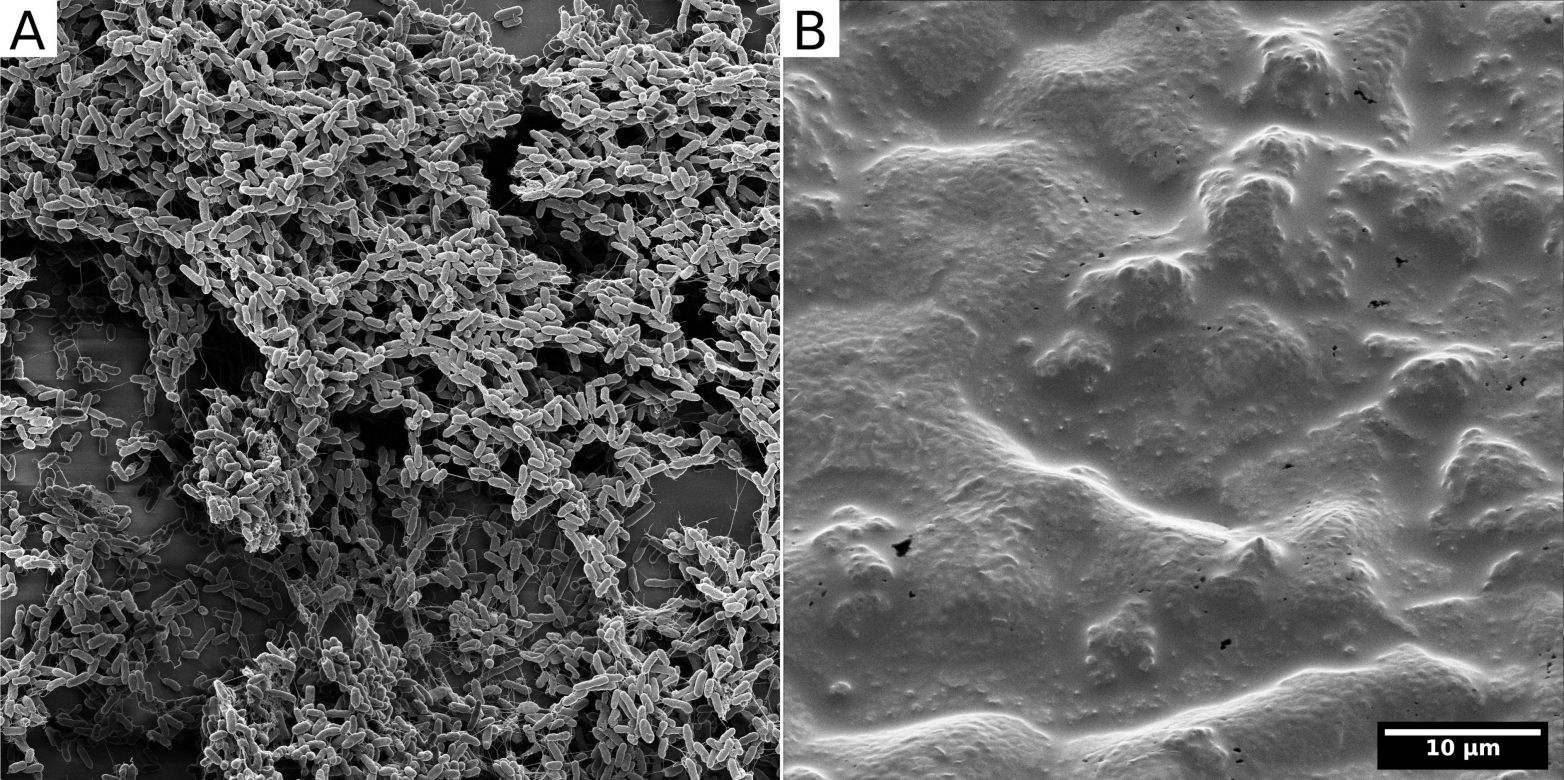
HIM of two *Pseudomonas putida* biofilms grown on polyvinylchloride coverslips in parallel under exactly the same conditions. Both films were chemically fixed with 2% glutaraldehyde for 2 h at room temperature. Subsequently (A) was dehydrated in a graded ethanol series and dried with hexamethyldisilazane. In contrast, (B) was not dried but the water was substituted with the ionic liquid 1-butyl-3-methylimidazolium tetrafluoroborate according to the protocol published by Golding et al. [69]. Unpublished data, sample provided by Nedal Said.

An alternative approach to the fixation, dehydration, drying, and even coating stages outlined above is to apply ionic liquids during sample preparation. Ionic liquids are organic salts with low melting points which are fluid at room temperature. They are persistent as liquids under the high-vacuum conditions of a typical electron microscope and exhibit conductive properties. This means that samples can be immersed in an ionic liquid, for 10–600 s, blotted, loaded onto a sample holder, and then imaged [68]. Compared to other preparation techniques, the preparation time using ionic liquids is extremely short and the morphology is reasonably well maintained. However, the surface is less well preserved. Figure 4 shows a *Pseudomonas putida* biofilm imaged using HIM [69]. One image shows the biofilm prepared using ethanol dehydration followed by HMDS drying (Figure 4A). The other image shows the biofilm prepared with an ionic liquid (Figure 4B). Though the HMDS-prepared sample clearly shows a high density of individual bacteria, the ionic liquid treatment appears to have maintained the EPS and is perhaps a better representation of the true biofilm. Nevertheless, this study suggests that multiple preparation treatments should be applied to best image a sample.

### HIM in medical research

Besides the example micrograph of a white blood cell published by Ward et al. in 2007 [2], the usage of HIM for medical studies did not begin until around 2011 when Arey et al. reported on the interaction of alveolar epithelial cells with silica nanoparticles with HIM at the Microscopy and Microanalytics Conference 2011 [11]. In their abstract, they suggested that Rutherford back-scattering imaging in the HIM enables the distinction of nanoparticles from cell surface structures at nanometre resolution. In a different study published in the same year, Bazou et al. employed HIM to image human colon cancer cells (Caco2) [9]. The glutaraldehyde-fixed and freeze-dried cells were imaged by both HIM and SEM to enable the direct comparison between the two instruments. HIM analysis of gold-coated and uncoated samples showed that coating, as required for SEM, introduces artefacts such as a granular structure on the cell surfaces and a partial closing of pores, thus highlighting one of the benefits of HIM. Bazou et al. also studied tumour cell-induced platelet aggregation by fluorescence microscopy and HIM. In this study, HIM provided high-resolution insight into the complex network of interactions of platelets with cancer cells [10].

**Figure 5:**
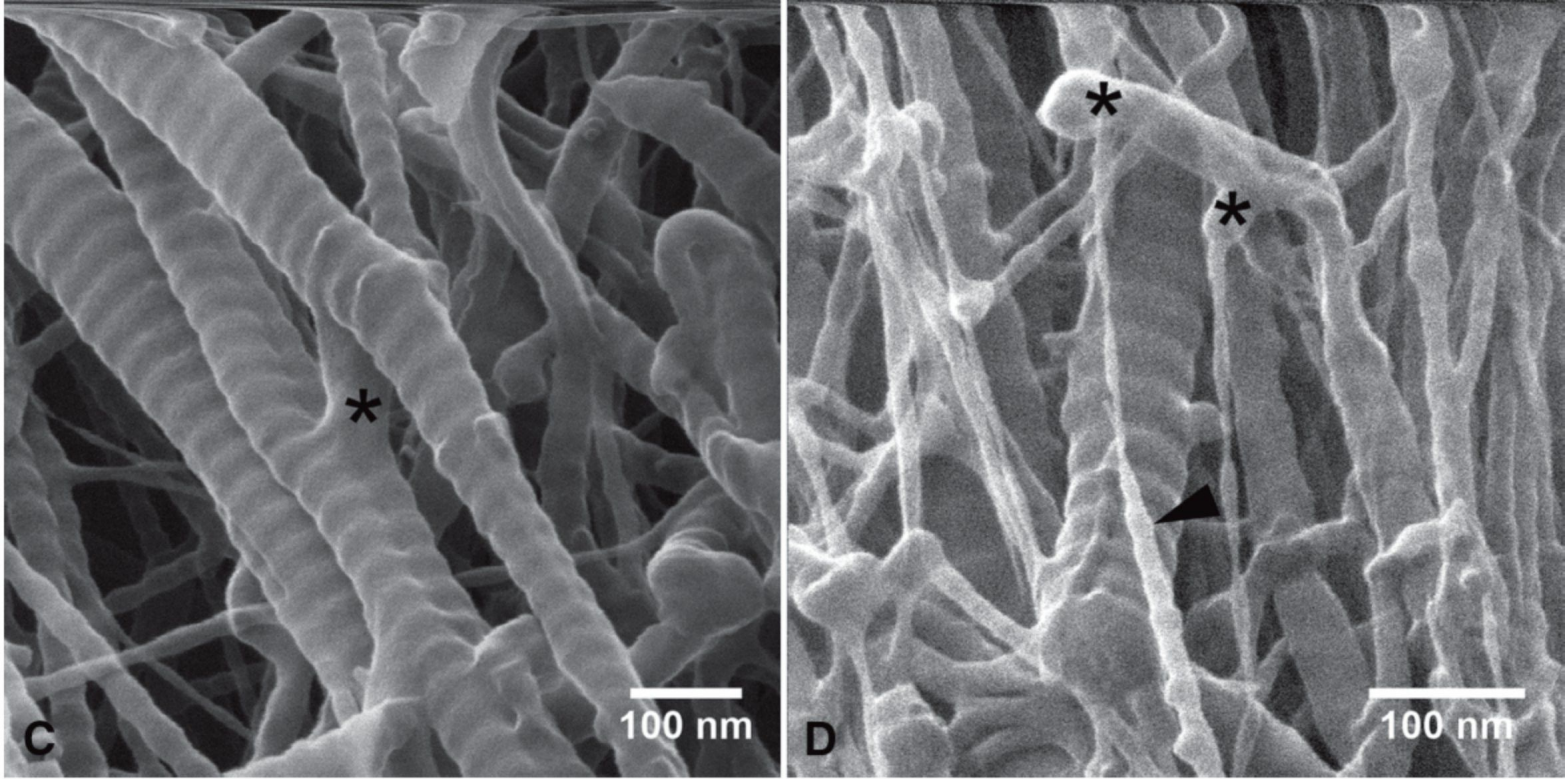
Rabbit cartilage collagen imaged by HIM. High resolution and depth of field reveal nanoscopic network features such as nanofibrils and their connections (asterisks). Dwell time and resolution are, respectively, 2 μs, 0.90 nm (left) and 0.5 μs, 0.81 nm (right). Reproduced from [70]. Copyright ©2012 The Authors Journal of Microscopy; ©2012 Royal Microscopical Society. Used with permission from Vanden Berg-Foels et al., Helium ion microscopy for high-resolution visualization of the articular cartilage collagen network, Journal of Microscopy, John Wiley and Sons.

In 2012, Berg-Foels et al. used transmission electron microscopy, SEM, and HIM to image rabbit cartilage samples [70]. The long depth of field provided by HIM renders the technique particularly powerful for imaging the three-dimensional articular cartilage collagen networks at a resolution of down to 0.81 nm (Figure 5).

Another application benefiting from the large depth of field and the possibility to work without coating the sample with metal is the use of HIM for the investigation of the development of mammalian tooth enamel [67,71]. Tooth enamel consists of hydroxyapatite crystals, which form needle-shaped nanocrystals of several micrometres length with a diameter below 100 nm. HIM revealed insight into the complex interactions between the enamel-forming cells, matrix proteins, and the mineral phase [71]. Bidlack et al. visualised the amelogenin proteins involved in tooth enamel development in a study combining SEM and HIM for three-dimensional imaging [67].

**Figure 6:**
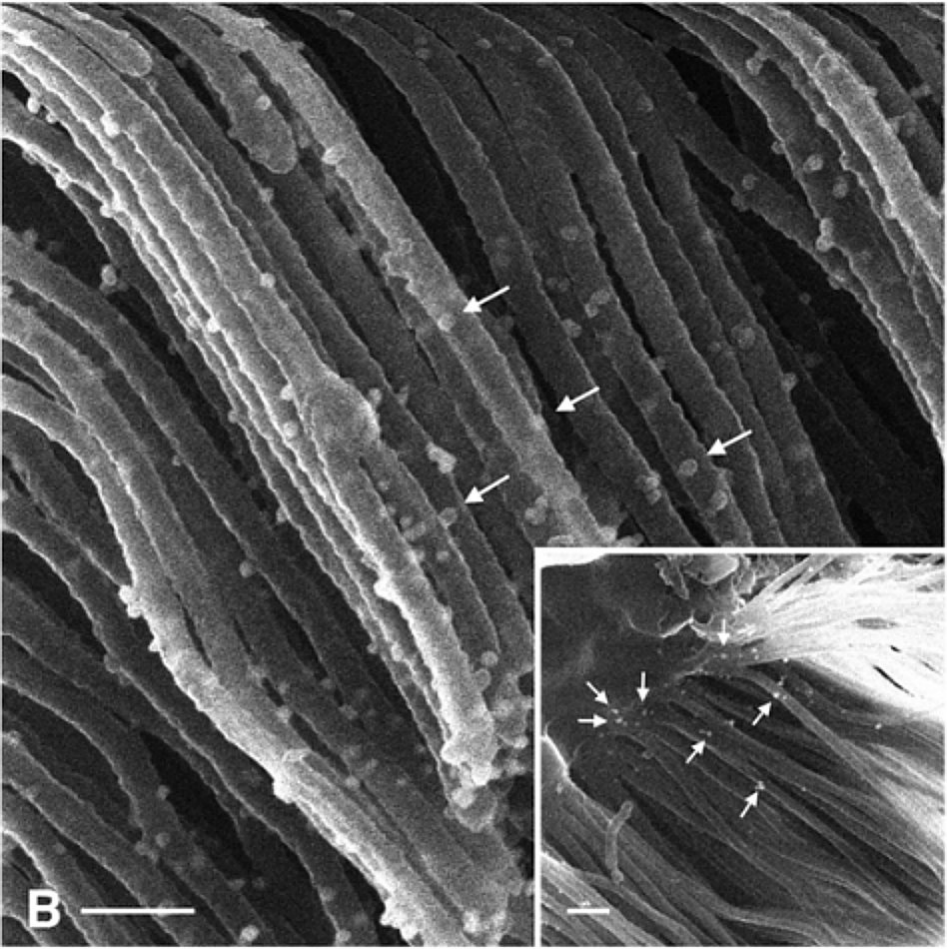
Helium-ion micrograph showing an immunogold-labelled (arrows) proximal tubule in a mouse kidney. Scale bar is 200 nm. Reproduced from [15]. Copyright ©2013 Rice et al., distributed under the terms and conditions of the Creative Commons Attribution License CC BY 4.0., https://creativecommons.org/licenses/by/4.0/.

In 2013, Rice et al. presented the, to the best of our knowledge, first images of immunogold labels detected by HIM [15]. For this study, the authors used the proximal tubule marker gp330/megalin and wheat germ agglutinin to label surface glycoproteins of the proximal tubule in mouse kidneys. Conjugation to 26 nm colloidal gold allowed the authors to visualise the label with the HIM (Figure 6). Soon afterwards, a number of publications on HIM imaging of kidneys were published. Paunescu et al. focused on the microstructures of the kidney glomerulus as well as on the brush border microvilli of the proximal convoluted tubules [72]. On the latter they found “micropits on the microvillar surface as well as thin filaments joining adjacent microvilli” at high magnification. Tsuji et al. investigated the alteration of endothelium and podocytes during progressing glumerulopathy in Col4a3 (Alport syndrome) mice [73]. HIM was used to visualise the podocyte and endothelial interface, which, in contrast to previously published transmission electron micrographs of sections, provided pseudo-3D data. The authors pointed out that using conventional SEM it was not possible to determine whether the glomerular basement membrane defect affects the endothelial structure. However, they stated “HIM allows the endothelial surface to be directly and clearly visualised.”

Using HIM, Schürmann et al. impressively showed how closely ultrastructural details of cell membranes visualised by HIM are linked to sample preparation [16]. In the study, lipid nanodomains in mammalian cells were investigated. HIM was used to image osmium tetroxide-fixed, uncoated, and critical-point-dried human neurons and mouse hippocampal neurons. The achieved lateral resolution of 1.5 nm allowed for visualising pits in the ultrastructure of the cell membranes. Based on that finding the authors hypothesised “that the pit-like domains are a direct visualization of the shape of membrane nanodomains, including lipid rafts and caveolae.” It was concluded that the pits result from the sample preparation since “the cell fixation with OsO_4_ cross-links the lipid bilayer outside the nanodomains while the lipid bilayer inside the nanodomains is removed by the required subsequent rinsing with ethanol for the cell drying process.” In turn, the pits in the HIM image reveal “the shape of the nanodomains as missing lipid bilayers.”

In 2018, HIM was used to study peptide nanostructures for the first time. Herrera et al. studied the initial stages of the oligomerisation of the 33-mer peptide gliadin [74]. The HIM data helped the authors to “show a plausible pathway of 33-mer peptide protofilaments formation” via the contact of square-like oligomers and the formation of protofilaments by “longitutinal association of matured rod-like oligomers.”

### Imaging animals and plants

**Figure 7:**
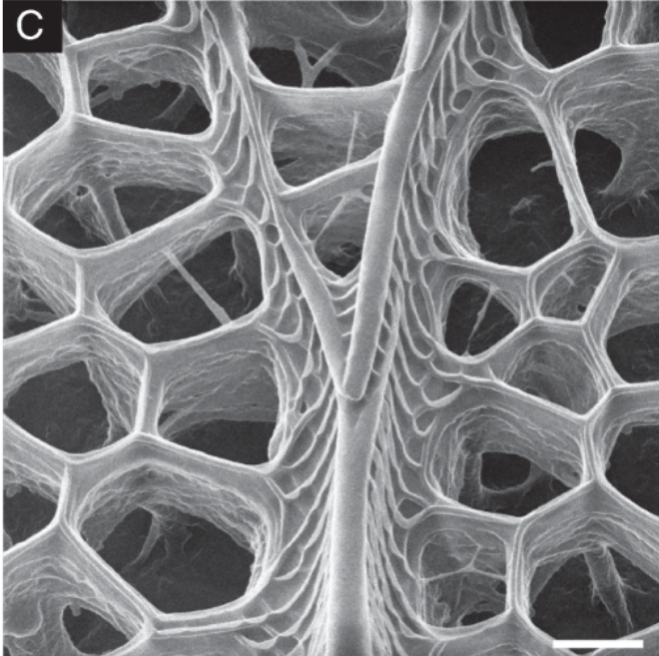
HIM image of a *Papilio ulysses* butterfly black ground scale. Scale bar is 400 nm. Adapted from [12]. Copyright 2011 ©Wiley Periodicals, Inc. Used with permission from Boden et al., Helium ion microscopy of Lepidoptera scales, Scanning, John Wiley and Sons.

HIM imaging of small animals and plants has been around since the early years of helium ion microscopy, when pollen samples [7–8] and pine leaves [14] were imaged. The first in-depth and notable HIM imaging demonstration of small animals was done in 2012 by Boden et al. [12], when the intricate micro- and nanostructures responsible for the structural colouration of the wings of two different butterfly species, *Papilio ulysses* (Blue Mountain Butterfly) and *Parides sesostris* (Emerald-patched Cattleheart), were imaged to a level of detail not obtained previously with SEM. The study took advantage of the strengths of HIM producing images with a large depth of field and a high level of surface detail (Figure 7). The work also directly demonstrated the superiority of HIM at high magnifications over environmental SEM, the older technology for high-resolution microscopy of uncoated insulating samples. In addition, the large depth of field of the HIM was exploited in an innovative way by creating stereo pairs of images.

Fairly soon after applying HIM on butterflies, HIM was also used for ultrastructural analysis of both wild-type and genetically modified fruit flies, *Drosphila melanogaster*, by Boseman et al. [13]. Many different areas were imaged such as the eye, the wing and body surfaces, the sensory bristles, and the legs, with observations of nanoscale features. In addition, the pupal case and some larval tissues were also investigated.

In 2013, Joens et al. [6] published a groundbreaking study regarding biological HIM imaging of a whole variety of biological samples, including plants, bacteria, cancer cells, and a nematode worm, *Pristionchus pacificus*. The imaging of that worm will be discussed later in the section “Nanofabrication” regarding its innovative use of the combination of milling and imaging. Plant imaging was done on the model species *Arabidopsis thaliana*. The HIM images of the uncoated cuticle samples showed fine textures and minute ridges not discernible in the low-voltage field-emission SEM images of the same samples. *Arabidopsis* samples were also HIM-imaged by Curtin et al. [75]. Their study examined how the surface texture of genetically different samples varied after acid treatment to see the potential for enzymatic biofuel production.

HIM imaging was once again used in studies of insect wings and their nanostructures by Bandara et al. [76–77]. In this case, a nanopillar texture on the wing of a dragonfly *Orthetrum villosovittatum* was studied. In addition to imaging the wing alone, samples were prepared with *E. coli* bacteria on them, to study the bactericidal properties of the nanostructure. Along similar lines, the nanostructures on the wings of three different species of *Cicada* were imaged using HIM in [78].

### Microbiology

HIM imaging has already been used to study numerous microorganisms including bacteria [6,18–19,60,79–80], bacteriophages [17–18,81], yeast [82], protozoa [83–85], and microalgae [46]. The highly resolved and contrast-rich views of the tiniest creatures that were obtained in these studies are discussed in the following.

#### Virus particles/bacteriophages

Bacteriophages are viruses that use bacteria as hosts, often causing lysis of the bacterial cell at the stage where new virus particles are released from the cell. For this reason, they have long been considered as possible treatments for bacterial infections. In particular, because they are host-specific and, therefore, do not exhibit some of the side effects of the more broadly affecting chemical antibiotics, which can lead to evolution of strains with broad antibiotic resistance.

Microscopy of bacteriophages has a long history, going all the way back to 1940, as one of the very first imaging applications of the newly invented electron microscopes (for a review see [81]). Ever since, TEM has been the mainstay of phage microscopy, but the complexities of TEM and SEM sample fabrication (lamellae preparation and conductive coatings) have hindered the studies of phage–bacterium interactions in their natural microbial environments.

**Figure 8:**
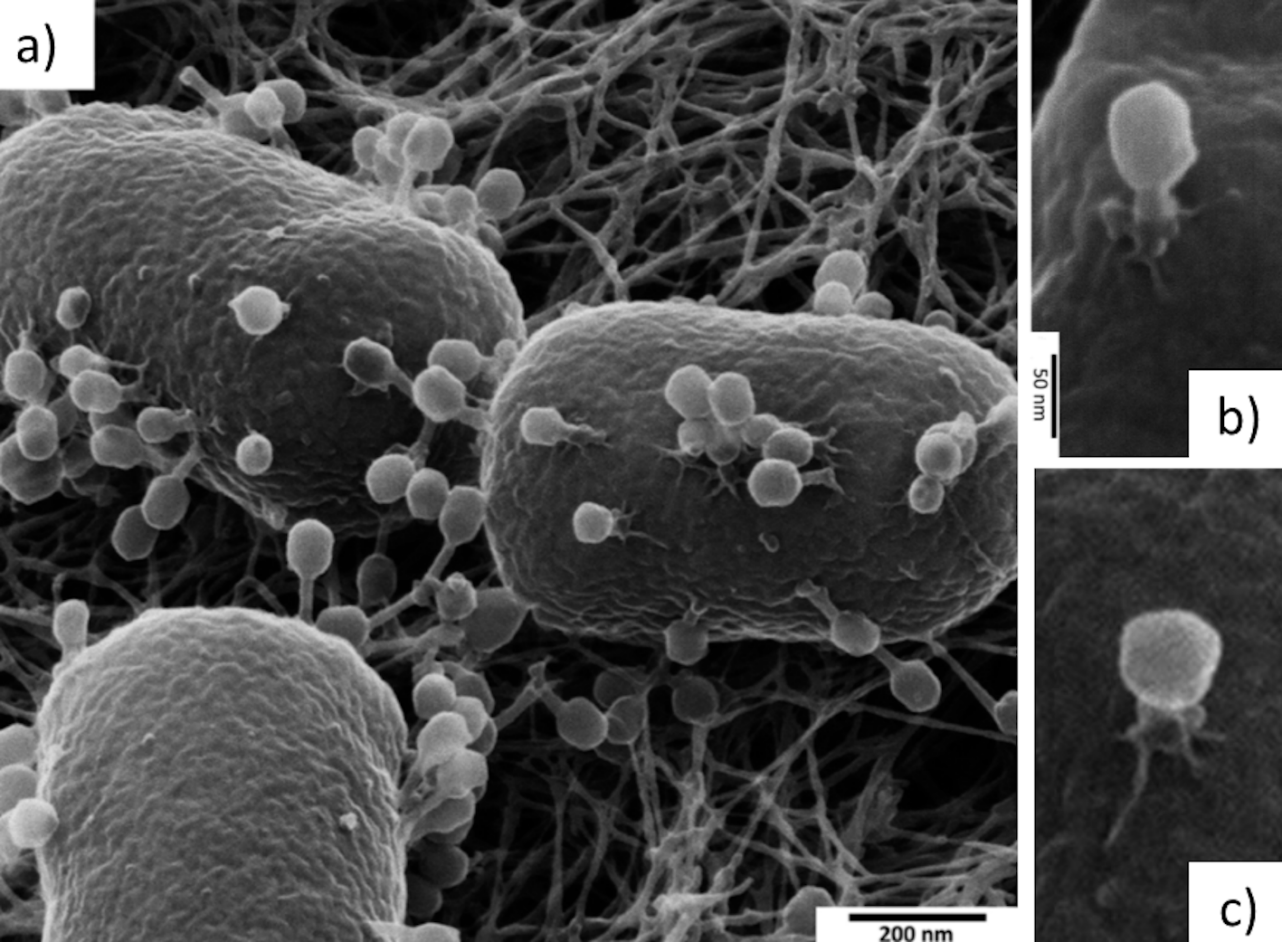
Helium-ion micrographs of the T4 bacteriophage infecting *Escherichia coli*. (a) Three bacteria with ongoing infection. (b) A higher-resolution image of a single T4 bacteriophage attached on the cell surface. The tail is contracted and the tail fibers are spread out, indicating a genome injection in progress. The icosahedral shape of the head is also apparent. (c) Another individual phage with even more contracted tail. Adapted from [17]. Copyright ©2017 WILEY-VCH Verlag GmbH & Co. KGaA, Weinheim. Used with permission from Leppänen et al., Imaging bacterial colonies and phage-bacterium interaction at sub-nanometre resolution using helium-ion microscopy, Advanced Biosystems, John Wiley and Sons.

HIM imaging of phages and phage–bacterium interactions were performed for the first time in 2017 in [17] for bacterial colonies of *E. coli* on an agar substrate. Different stages of the phage life cycle were imaged by looking at different regions of the viral plaques caused by the initial T4 phage infection seeded at the centre of the plaque. Figure 8 shows some examples of the detail obtained in this study. In particular, the changes in the appearance of the phage during active infection (contraction of the tail and spread-out of the tail fibres) were imaged.

Only a few works followed after this initial demonstration of the HIM capabilities for bacteriophage studies, although there is clearly a potential to image many more types of phage–bacterium systems. One more recent example was in a study by Sharma et al. [18] in which environmental sediment samples were imaged using HIM with findings of viruses attached to bacteria.

For bacteriophage imaging, the strength of HIM lies not just in the resolution, which is higher than that of SEM, but in the possibility to study the phage–host interaction, with a sufficiently high resolution to see nanometre-scale details of the phage particles (Figure 8).

#### Predatory bacteria

Similar to bacteriophages, predatory bacteria are bacterial parasites. In contrast to bacteriophages, predatory bacteria have a metabolism and undergo cell division for reproduction. However, compared to other bacteria, metabolism and reproduction are very uncommon and require preying on other gram-negative bacteria [86]. Therefore, following the philosophy of “the enemy of my enemy is my friend”, bacterial predators are promising candidates for “living anti-biotics” [87]. While bacteriophages are very host-specific, *Bdellovibriones* can be considered as broad-spectrum antibiotics, as they can potentially infect all gram-negative bacteria. Considering the 2017 priority list of antibiotic-resistant bacteria published by the World Health Organisation, the targeted use of *Bdellovibrio* would be a strong means to fight the three highest-priority strains, which are all gram-negative [88].

**Figure 9:**
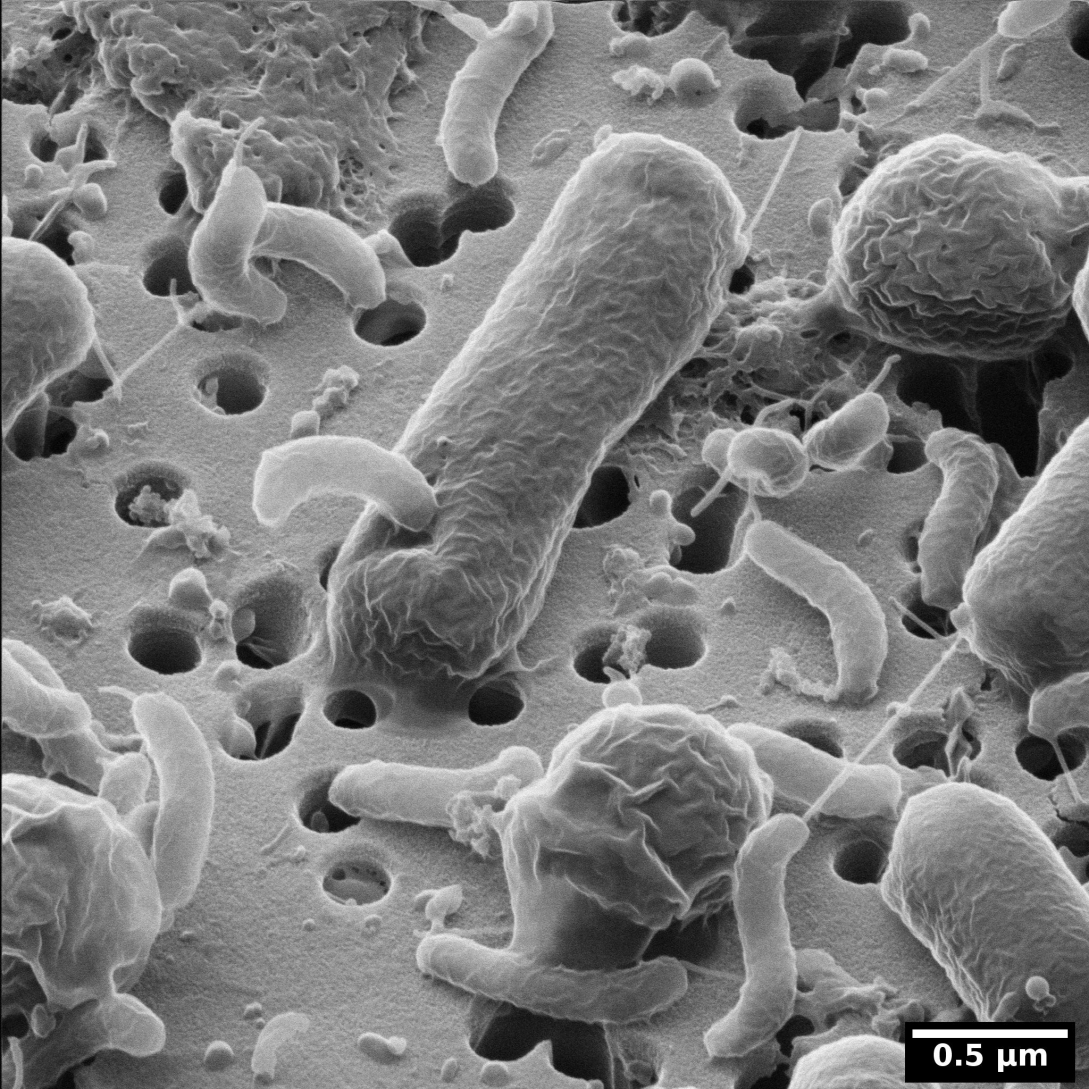
Helium-ion micrograph of the predatory bacterium *Bdellovibrio bacteriovorus* infecting *Escherichia coli*. The sample was prepared by M. Krüger and N. Said as preliminary work for the study published in [19].

Said et al. investigated the life cycle of the bacterial predator *Bdellovibrio bacteriovorous* HD100 with the HIM [19]. At time *t* = 0 they combined cultures of *Bdellovibrio* and prey, for example, *Escherichia coli*, and stopped the experiment by chemical fixation at specific points during incubation. This allowed for the study of the attachment of the predator to its prey, followed by penetration of the membrane, and the entering into the cytoplasm. After that, the prey cell is transformed into a bdelloplast, in which the predator elongates and divides. In a final step, the bdelloplast lyses and the *Bdellovibrio* offspring is released and ready to attack another cell. A HIM micrograph of the attachment of a *Bdellovibrio* to *E. coli* is shown in Figure 9. Again, it is not merely its high resolution but rather surface sensitivity and charge compensation that make HIM an excellent tool to study interactions of bacterial predators with their prey.

#### Eukaryotic parasites

**Figure 10:**
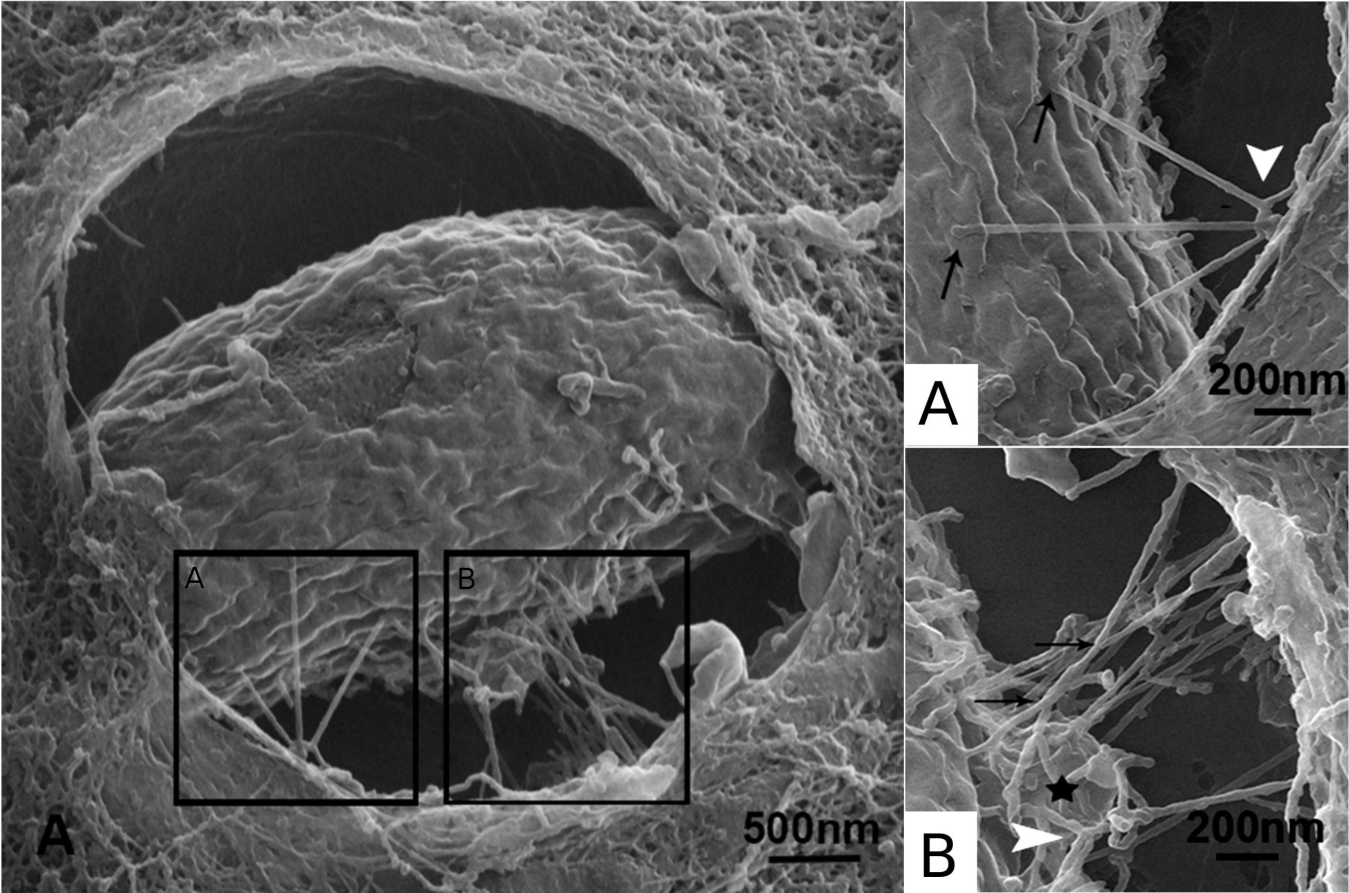
HIM of *Toxoplasma gongii* inside a vacuole of an infected Rhesus monkey kidney epithelial cell. The close-ups (A) and (B) show the intravacuolar network of tubules formed by the parasite soon after invasion. White arrows point at bifurcating tubules, black arrows point at crossing tubules that do not fuse. Adapted from [83], Journal of Structural Biology, Vol. 191(1), by de Souza et al., “New views of the Toxoplasma gondii parasitophorous vacuole as revealed by Helium Ion Microscopy (HIM)”, pages 76–85, Copyright (2015), with permission from Elsevier.

Unicellular parasitic eukaryots have also been imaged using HIM. In 2015, de Souza and Attias investigated *Toxoplasma gongii*, an obligate intracellular parasite which causes the disease toxoplasmosis [83]. Extracellular *Toxoplasma gongii* are found to be “teardrop-shaped” with an apical conoid. Rhesus monkey kidney cells were infected with *Toxoplasma gongii*. After dry-cleaving, parasitophorous vacuole in the cells were exposed and the intracellular parasite was imaged. The helium-ion micrographs revealed an intravacular network of tubules formed by *Toxoplasma gongii* (Figure 10).

In the same year, Gadelha et al. investigated *Giardia intestinalis*, a flagelled parasite causing the diarrheal disease giardias, with ultrahigh-resolution SEM and HIM [84]. Of particular interest to the authors was the cytoskeleton of *Giardia intestinalis* for which HIM enabled the visualisation of “a lattice-like array material that covered the microtubular sheets of the funis.” A review article on protozoa imaging by de Souza and Attias nicely placed HIM imaging in the context of high-resolution SEM, environmental SEM, cryo-SEM, the usage of cyto-chemistry, and 3D reconstruction with focused ion beam SEM and TEM [85].

#### Biofilms

The large depth of field, the efficient charge compensation and the strong edge contrast make HIM an excellent tool for the visualisation of the structural organisation of biofilms. To date, the great potential of HIM for the analysis of biofilms has not been fully exploited yet and only few publications exist. To our knowledge, the earliest publication in this direction is the extended abstract of LeTourneau et al. on rhizobacterial biofilms in the proceedings of Microscopy and Microanalytics 2015 [89]. On wheat roots, they grew a phenazine-1-carboxylic acid (PCA)-producing fluorescent pseudomonad strain and an isogenic mutant impaired in the synthesis of PCA. The formation of the rhizobacterial biofilm on the root under dryland and irrigation conditions was studied by fluorescent microscopy and SEM on a larger scale. HIM was used to visualise the nanostructure of the bacterial colonies; however, no micrographs with sub-20 nm resolution are shown. The second publication is an extended abstract by Belianinov et al. on the investigation of the biofilm structure of *Geobacter sulfurreducens* by HIM [79]. The helium-ion micrograph of the bacterium shown in this abstract displays the importance of an appropriate preparation of the sample. The preparation method used by the authors – chemical fixation in 4% formaldehyde, rinsing in buffer, dehydration with ethanol, and air-drying – did not suffice to maintain the ultrastructure of the cell membrane as well as the fine layer of EPS, which HIM can, in principle, visualise.

**Figure 11:**
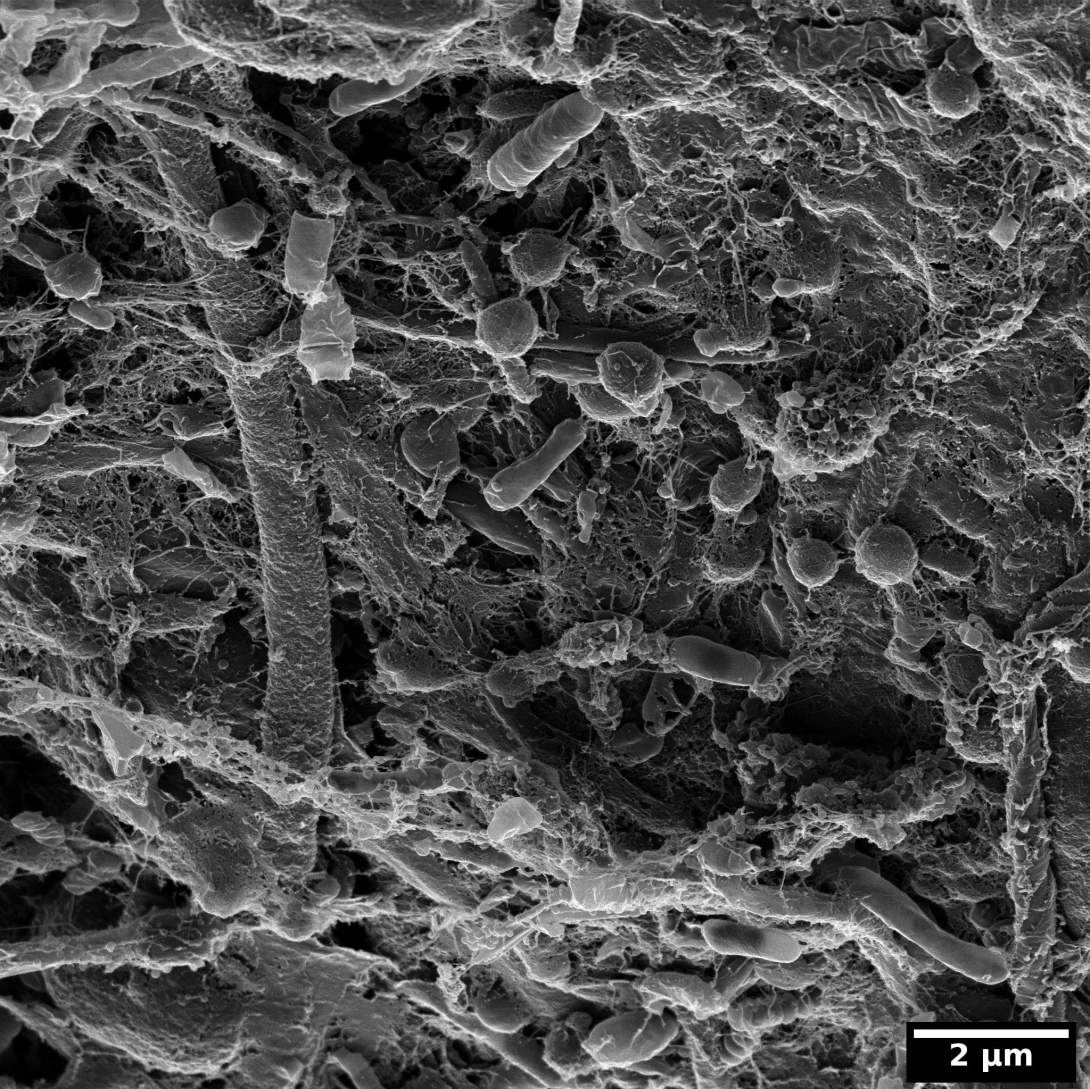
Microbial mat collected at the Himalayan hot springs at Manikaran imaged with the HIM. Unpublished data from the study of A. Sharma et al. [18].

In the context of the microscopic analysis of bacterial and archaeal viruses of the Himalayan hot springs at Manikaran, Sharma et al. [18] imaged microbial mats with the HIM (Figure 11). The variety of microbes embedded in EPS shows the complexity of natural biofilms. Preparing these samples for HIM is difficult because each fixation, rinsing, dehydration, and drying step is a trade-off between preservation of the cell morphology, avoiding precipitates on the sample and maintaining the filigreed EPS. Therefore, Sharma et al. used a protocol both simple and effective: The sample was kept in the medium (water from the hot springs at Manikaran) to which gently and slowly ethanol was added. Once a concentration of about 70% ethanol was reached, the sample was kept in the fridge overnight in order to use the gentle fixation effect ethanol provides [90]. Subsequently, the ethanol/medium mixture was pipetted off and the sample was treated with a graded ethanol series to finish the dehydration. After critical point drying the sample was ready for HIM analysis.

**Figure 12:**
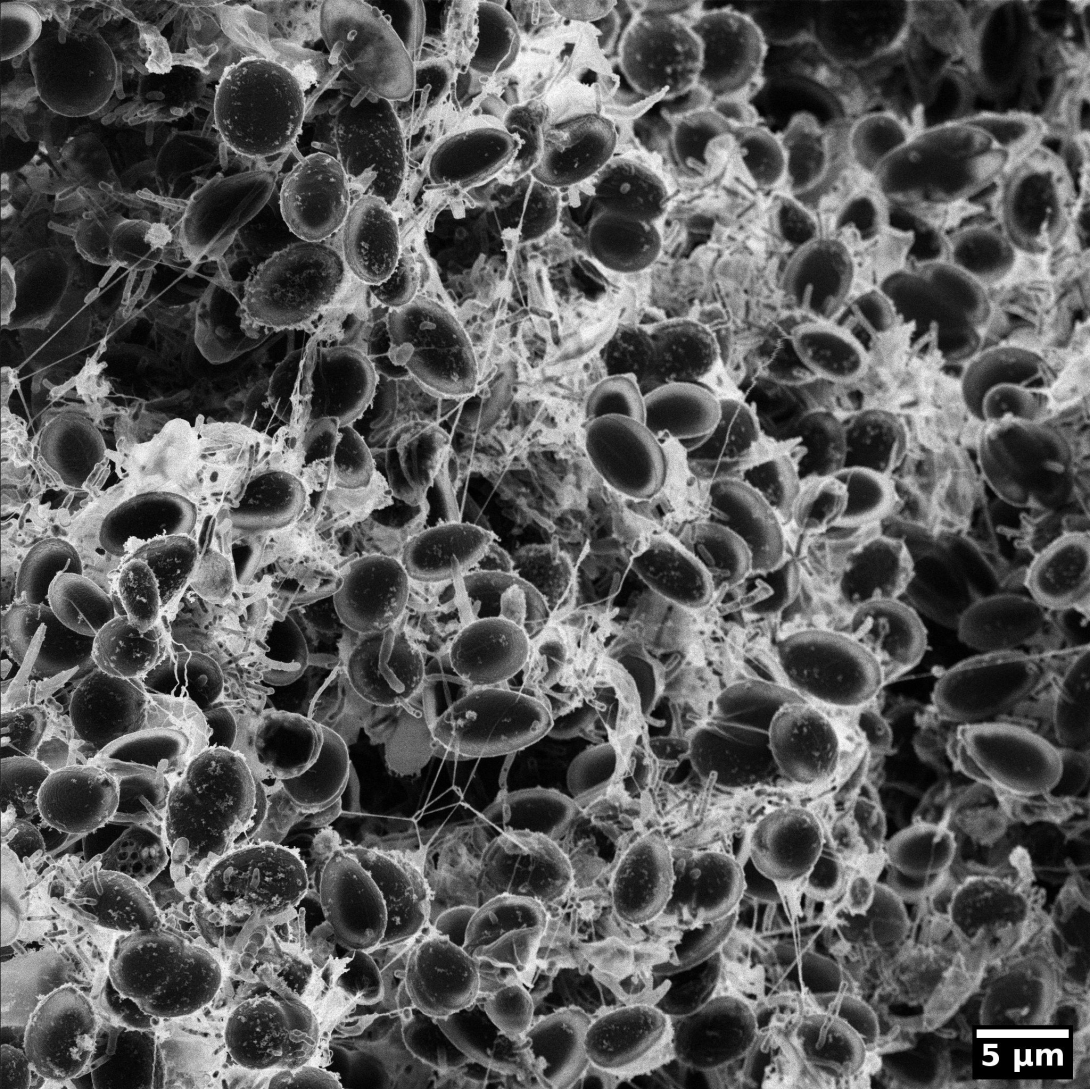
A biofilm of *Chlorella* microalgae imaged using HIM. Charge compensation allowed for imaging the biofilm without any metallisation. The exopolymeric polymeric substances between the algal cells are visualised with high contrast owing to the high surface sensitivity of the HIM. The sample was prepared by J. H. Moreno-Osorio within the study published in [46].

Moreno-Osorio et al. [46] investigated biofilms formed by *Chlorella* microalgae of which an example is shown in Figure 12. In this study, the biofilm was fixed in 4% paraformaldehyde dissolved in sodium cacodylate buffer for 2 h at room temperature, followed by rinsing in buffer to remove precipitates. Subsequently, the sample was dehydrated in a graded ethanol series and critical point dried. This approach results in an excellent preservation of the algal cells, however, at the cost of a partial shrinkage of the EPS. The micrograph was recorded using secondary electron imaging. The high surface sensitivity and the strongly pronounced edges in this imaging mode render the thin EPS between the algal cells bright white. Furthermore, it is remarkable that despite the strong topography of more than 20 μm in the field of view, almost the entire image is well focused owing to the large depth of field of the HIM.

### Geomicrobiology

Microbe–mineral interactions are fundamental to many processes taking place in the environment such as rock weathering, nutrient release, toxic metal(loid) mobilization, and greenhouse gas formation [91]. However, the combination of soft biological material with hard minerals brings unique challenges to imaging these interactions at nanoscale resolution. Traditionally, scanning electron or transmission electron microscopy techniques have been applied to great effect to study many aspects of microbe–mineral interactions, such as the formation of intra- or extracellular mineral precipitates, or intracellular organelles associated with energy gain or electron transfer [92]. However, over recent years, the number of articles related to geomicrobiology and containing HIM data has increased. This is due to several factors such as the ability to image without first coating a sample with conductive materials (e.g., Pt, Au, or C). Such coatings have been shown to cause artefacts which can be misinterpreted as being from the material under investigation [6]. Furthermore, helium ions have a smaller interaction volume with a sample than electrons. This means that HIM can be used to provide better material contrast and depth of focus compared to SEM. Consequently, HIM is considered as a viable alternative to SEM for studying processes in geomicrobiology, with a steadily increasing number of published articles reflecting this fact.

**Figure 13:**
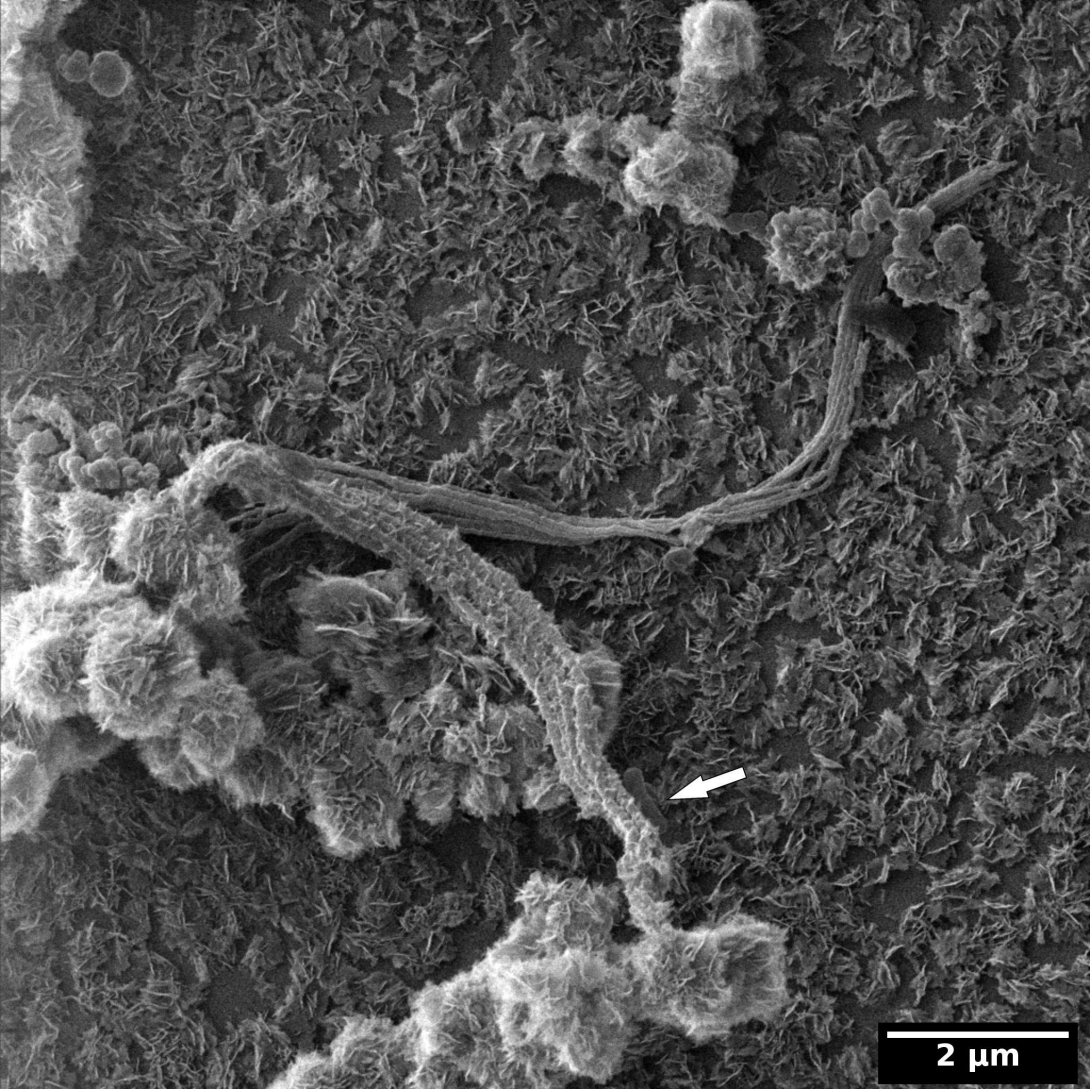
Helium-ion micrograph of a twisted stalk produced by a microaerophilic Fe(II)-oxidizing bacterium (unpublished). The white arrow indicates a single bacterium.

One area which has seen increasing use of HIM is the investigation of redox interactions between iron-metabolizing bacteria with ferrous (Fe(II)) and ferric (Fe(III)) iron. The ubiquity and availability of iron on Earth has led to the evolution of Fe(II)-oxidizing and Fe(III)-reducing bacteria, which use Fe(II) and Fe(III) as electron source and sink, respectively. Three types of Fe(II)-oxidizing bacteria are thought to exist under neutrophilic conditions in the environment including phototrophs, microaerophiles and nitrate-reducers [93]. Phototrophic Fe(II) oxidizers use light as energy and Fe(II) as an electron source for growth. They are thought to have been partially responsible for the deposition of banded iron formations during the Archean. Laufer et al. [66] isolated a halotolerant anoxygenic phototrophic Fe(II)-oxidizing green sulfur bacterium from a marine sediment and used HIM to show the surface of the cells to be smooth and free of iron minerals. This lack of cell-surface encrustation is considered to be an important identifier. Byrne et al. [80] used HIM to investigate the formation of organic–metal fibres, known as twisted stalks, by microaerophilic Fe(II)-oxidizing bacteria. These stalks consist of organic material as well as nanometre- and micrometre-sized iron minerals which are loosely bound to the bacterial cells (Figure 13). It is thought that these appendages help to eliminate Fe(III) waste produced during Fe(II) oxidation and provide a surface for the sorption of nutrients as well as heavy metals. In the study, Byrne et al. took advantage of the flood gun to analyse the development of twisted stalks over time without coating the samples with a conductive material. They observed the precipitation of lepidocrocite plates, which appeared to grow and become denser over time. Regarding nitrate reducers, Joens et al. [6] published the earliest article using HIM to investigate microbe–mineral interactions. The authors compared the performance of both SEM and HIM for studying the nitrate reducer *Acidovorax sp.* BoFeN1. The authors noted the ability of HIM to greatly reduce charging artefacts associated with field-emission SEM. The same organism was also investigated by Zeitvogel et al. [60]. They investigated the effect of preparation approaches on sample preservation and how this affects the quality of the HIM micrographs (see section “Sample preparation”). Nordhoff et al. [94] applied HIM to investigate microbe–mineral associations for culture KS, which is another nitrate-reducing Fe(II) oxidizer and is in fact the only widely accepted bacterium which is able to enzymatically couple nitrate reduction with Fe(II) oxidation. To date, there are not many studies which have applied HIM to study microbial Fe(III) reduction. The study by Belianov et al. [79], who imaged a biofilm of the Fe(III)-reducing bacterium *Geobacter sulfurreducens*, is the only published example, to the best of our knowledge.

Apart from iron geomicrobology, a study by [95] used HIM to characterise the colonization of mineral substrates by the soil mineral-weathering microorganism *Pinus resinosa*, which is widespread in North American pine forests. Through correlative approaches with TEM, SEM, HIM, and X-ray tomography, they were able to gain insight into microbially driven weathering processes under nutrient-rich conditions. Mineral weathering was also assessed by [96] and [97], who developed methods to analyse biological and geochemical drivers of weathering in natural settings. HIM was used to image sub-nanometre mineral–organic interactions, whereas SEM (EDX) was used for elemental quantification. They preferred the superior imaging capabilities of the HIM to image minerals weathered in soil mesh bags filled with granular granite, basalt, and quartz (53–250 μm), which were deployed in the field for one year. They were able to identify grain microtopography and nanocrystal edges characteristic of quartz, lamella structures and smooth surfaces of biotite in the granite, and vesicles of basalt embedded with amorphous glass. The HIM also revealed a fungal hyphae network in all samples with nanoscale imaging suggesting accelerated weathering along the mineral surface due to biological interactions. Moreover, HIM is being applied for soil-based studies. Biochar, a carbon-rich material formed by the pyrolysis of biomass is under fervent research because of its ability to improve soil quality and improve agricultural productivity. Rasa et al. [98] used HIM to investigate the relationship between internal porosity and pore size distribution of biochar. HIM indicated that cell-wall structures in biochar do not contain visible nanoscale pores and suggest that the water storage and flow within willow biochar takes place in cylindrical capillaries. LeTourneau et al. [99] used SEM, HIM, and NanoSIMS to show that phenazine-1-carboxylic acid, produced by rhizobacteria in unirrigated wheat fields, and soil moisture promotes biofilm formation at root surfaces.

With the exception of Joens et al., all other geomicrobiology-related studies using HIM have been published in the past three years. Most recently, HIM was used to great effect, such as for studying vesicular structures budding off an ethane-degrading anaerobe (*Candidatus Argoarchaeum ethanivorans*) [100]. Overall, it is thus clear that HIM is an extremely powerful technique and is likely to be used increasingly in the field, especially, if analytical capabilities soon become more widespread.

### Nanofabrication

Ion microscopy has the added benefit over electron microscopy that the same instrument has the capability not only to image, but to also mill away parts of the sample material. The change between the two functionalities depends on the ion current and ion species. With sufficiently high currents, even He ions can mill materials. In recent years, another ion species also became available as an option in the commercial instruments, namely neon. As Ne is much heavier than He, it has a stronger milling capability, with the trade-off of reduced resolution. Initially, He-ion milling with HIM was used in non-biological applications such as the direct fabrication of graphene nanostructures and sub-5 nm size nanopores in suspended membranes. We will not review these applications in detail here, as they have been discussed in earlier reviews [101–102].

It is worth mentioning that some of the known nanopore applications are linked to biology, as nanopore membranes could be used for advanced DNA sequencing technologies or the filtration and detection of biomolecules. In contrast, much less work has been done regarding the milling of biological samples, which will be reviewed below.

**Figure 14:**
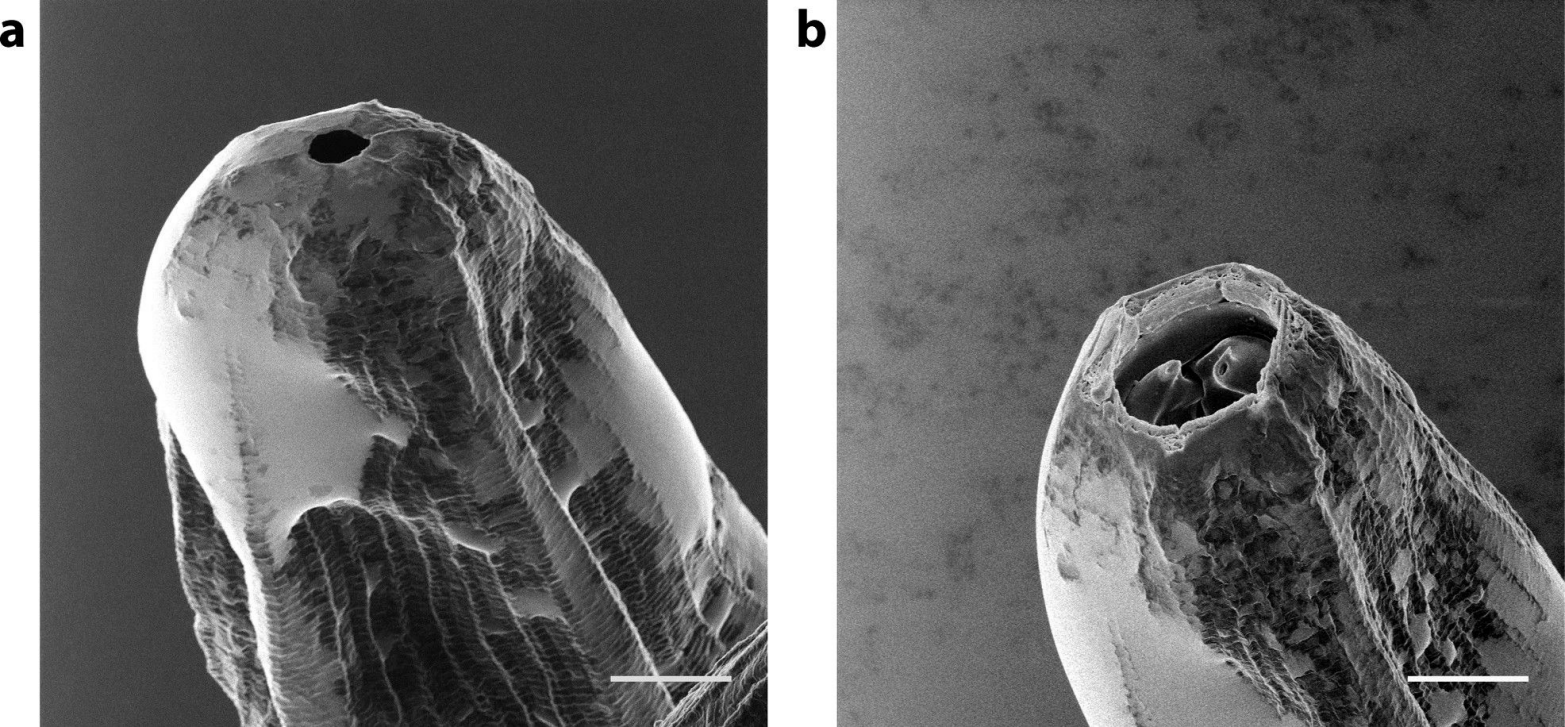
Helium-ion micrographs of the predatory nematode *Pristionchus pacificus* before (a) and after (b) the removal of the membraneous sheath covering the internal tooth structure by Ne-ion milling. Scale bar 5 μm. Adapted from [6], Joens et al., “Helium Ion Microscopy (HIM) for the imaging of biological samples at sub-nanometer resolution.” Licensed under a Creative Commons Attribution-NonCommercial-NoDerivs 3.0 Unported License, http://creativecommons.org/licenses/by-nc-nd/3.0/. Copyright © 2013, with permission from Springer Nature. This figure must not be reproduced or adapted without permission from Springer Nature.

One of the first practical examples of using the combined imaging and milling capabilities of a HIM in a biological study was demonstrated in the seminal work by Joens et al. [6], in which Ne-ion milling was used. They first imaged the mouth of a predatory nematode *Pristionchus pacificus* with HIM (Figure 14a) showing a membraneous sheath structure covering the internal mouth cavity. After that, the sheath structure was removed by milling, exposing the internal tooth structure, Figure 14b.

**Figure 15:**
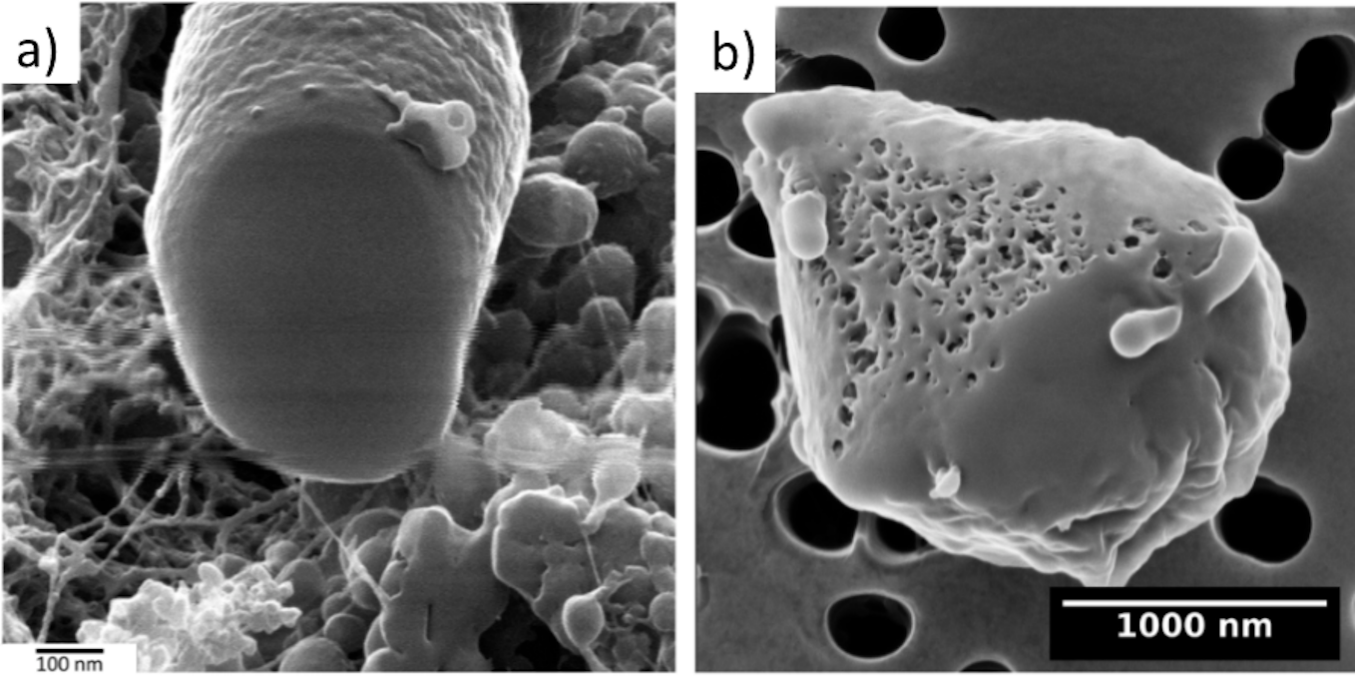
Helium-ion micrographs of sectioned microbiological samples. (a) He-ion-milled cross section of a *E. coli* with a half-cut bacteriophage on top of it. (b) Ne-ion-milled cross section of a *Bdellovibrio-E. coli* bdelloblast with visible internal structure and *Bdellovibrio* progeny penetrating the membrane. Figure 15a adapted from [17], Copyright © 2017 WILEY-VCH Verlag GmbH & Co. KGaA, Weinheim. Used with permission from Leppänen et al., Imaging bacterial colonies and phage-bacterium interaction at sub-nanometer resolution using helium-ion microscopy, Advanced Biosystems, John Wiley and Sons; Figure 15b adapted from [19], Copyright © 2018 WILEY-VCH Verlag GmbH & Co. KGaA, Weinheim. Used with permission from Said et al., Have an Ion on It: The Life-Cycle of Bdellovibrio bacteriovorus Viewed by Helium-Ion Microscopy, Advanced Biosystems, John Wiley and Sons.

Combining HIM imaging and He- and Ne-ion milling for microbiological samples has also been initiated. In the HIM-imaging study of bacteriophage–bacteria interactions by Leppänen et al. [17], cross-sectional He-ion milling of a *E. coli* cell was demonstrated (Figure 15a). Controllable cuts were made, but the resulting exposed surface was smooth and did not show any detail. In particular, no internal cavity or structure was found. However, the image does also show a cut bacteriophage attached on the surface of the bacterium, with a hollow head. For another species, *Flavobacterium* sp. 183 on Si substrates, some indications of cross-sectional details were also reported. No clear explanations for the differences of imaging detail between the two cases were offered, but one should at least note the very different substrates (porous dried agar vs solid Si), which could affect heat dissipation, for example. In the same study, a larger trench with 13 μm × 5 μm area and several micrometres deep was also milled into an agar substrate with Ne ions, exposing a subsurface bacterial colony.

In an another HIM-imaging study on a microbiological sample by Said et al. [19], Ne-ion milling was used to make a cross section of a *Bdellovibrio-E. coli* bdelloblast shortly after lysis (Figure 15b). A porous internal structure was revealed, with *Bdellovibrio* progeny penetrating the membrane. It is possible that the use of a Ne ion beam with its stronger cutting efficiency helped to reveal the internal structure better than in the milling study of Leppänen et al. [17].

In a recent study on the microencapsulation of bacteriophages with a membrane emulsification process, the internal structure of the microcapsules was also studied with a combination of Ne-ion cross sectioning and HIM imaging [20,103]. It was shown that the method of sample drying (either freeze drying or critical point drying) had a strong influence on the observed internal structure. However, it was not stated which preparation method represented the true structure.

**Figure 16:**
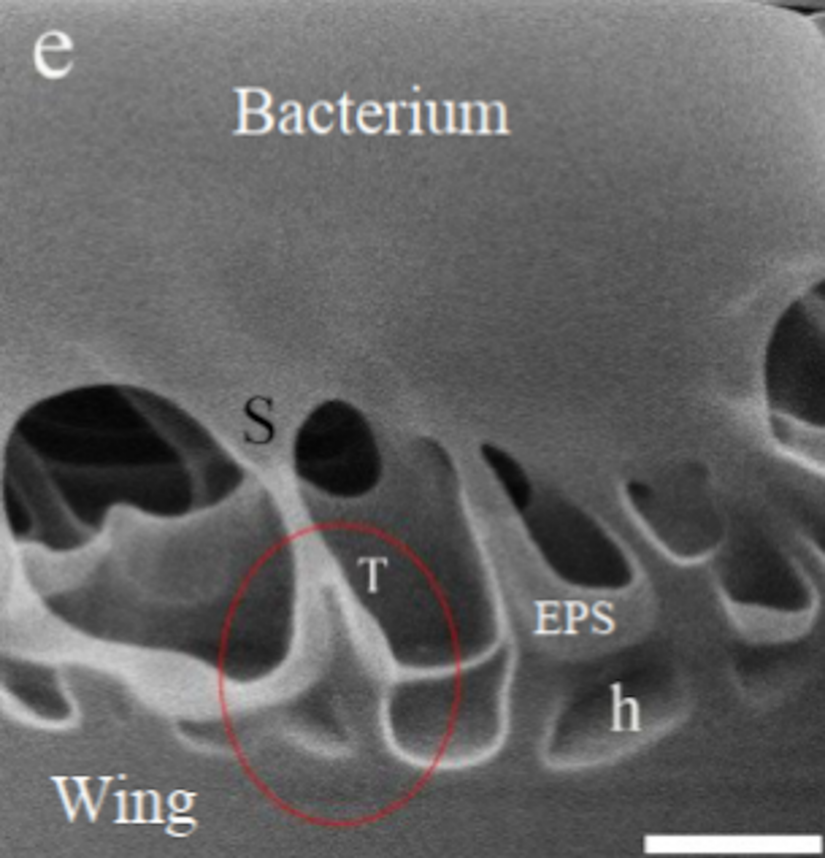
Helium-ion micrograph of a Ne-ion-milled section of a *E. coli*–nanopillared dragonfly wing interface. A partly deformed and stretched membrane is marked as “S”, and the tip of a nanopillar as “T”. Scale bar 200 nm. Adapted with permission from [104]. Copyright 2020 American Chemical Society.

Very recently, Ne-ion milling in HIM was also used to make very precise cuts of a *E. coli* bacterium on a nanopillared dragonfly wing [104], at the exact location where the bacterial cell is attached to the wing (Figure 16). The following HIM imaging revealed intricate sharp structures at the interface between the bacterium and the nanopillared wing surface. Clear deformations of the bacterial cell were visible, without any evidence of piercing of the cell membrane by the nanopillars.

All the above examples demonstrate that HIM milling combined with in situ HIM imaging is a promising novel avenue for the microscopy of subsurface structures and, sometimes, for cross sections. Internal porous structures are revealed particularly well. The strength of the technique is that the milling can be done in a point-and-shoot mode at a precisely determined location, in best cases at sub-nanometre resolution, without the typical and restricting resin-embedding and metal-coating techniques often used with focused gallium ion beam milling of biological samples in more common focused ion beam instruments.

### HIM in correlative approaches

The triad of imaging, nanofabrication, and nanoanalytics puts HIM amongst the most versatile microanalytical tools available at present. However, HIM alone cannot solve every problem in microscopy. In subcellular chemical imaging, for instance, correlative workflows combining high-resolution electron microscopy and nanoanalytics (e.g., X-ray spectroscopy and SIMS) are currently being established [105]. It can be expected that HIM imaging, HIM-SIMS, and IL-HIM will soon become part of such workflows because HIM will be one means to push the limits of nanoanalytics to the (sub-)10 nm range. Early work in this direction was published by Chen et al., who imaged human liver cells correlatively by HIM and fast (1.2 MeV) helium ions [106]. This correlative microscopy approach connected surface-sensitive HIM with He-ion energy loss data which represents the mass distribution in the cell. Furthermore, the authors speculate that ion-induced fluorescence will allow for fluorescence microscopy at the nanometre scale. Sanders et al. reported a correlative study on the interaction of rat cortical neural progenitor cells with gold nanoparticles at the Microscopy and Microanalytics Meeting 2014 [107]. They used HIM alongside with optical and fluorescense microscopy, electron microscopy, and electron-microscopic tomography techniques to locate cells and nanoparticles. Fluorescent markers were used to identify neurons and astrocytes, which subsequently were found and imaged at high resolution. The correlative studies by Sato et al. [25] and Moreno-Osorio et al. [46] have already been discussed in previous sections. Recently, LeTourneau et al. published a correlative study on rhizobacterial colonies on wheat roots where stable isotope labelling in combination with NanoSIMS, electron microscopy, and HIM, was used to study the carbon and nitrogen cycles [99]. The influence of phenazine-1-carboxylic acid and soil moisture on the biofilm formation by rhizobacteria was investigated. In the workflow of analyses, as can be expected, HIM was used to obtain structural information at high resolution. However, in future correlative workflows with HIM-SIMS or IL-HIM the role of HIM may change towards its nanoanalytical capabilities.

## Conclusion

The past decade of bio-imaging using HIM has seen the evolution of studies from those initially focused on the technological advantages of the instrument to more recent publications which use the HIM as part of a suite of tools to answer their research questions. This gradual change in the application of HIM provides an extremely positive indication that HIM has shifted from being highly specialised to being more widely applied and used to reveal information which could not have been previously obtained with more conventional techniques. The current generation of HIMs has remarkable capabilities in terms of spatial resolution, very large depth of field, and the ability to image non-conductive samples without the application of conductive coatings. These technical capabilities place the imaging performance of HIM well above the most technologically advanced SEMs. This is particularly evident for the ability to interrogate non-conductive samples, as is the case for most biological materials, without first sputter coating with a heavy metal, such as platinum or gold. This is possible thanks to the presence of the flood gun, which is, in effect, a charge-compensating device. It floods the imaging area with electrons to remove excess charge from a sample and to prevent localised build-up beneath the incident ion beam. Furthermore, the low energy transfer between helium ions and secondary electrons means that only the very near surface of a sample is imaged, similar to using an SEM with low acceleration voltage. In this way, HIM has been able to image the surface of biological materials as close to the native state as possible, without defects and artefacts which are evident when imaging a coated sample at high resolution or with low-voltage electrons.

On top of its remarkable imaging capabilities the ion beam of the HIM is one of the most precise tools for nanoscale fabrication. It has been already used for milling delicate objects such as bacteriophages [17], bdelloplasts [19], and bacteria on natural nanotextured surfaces [104]. However, the full potential of HIM nanoscale fabrication for biological applications has certainly not yet been exploited. The potential future applications comprise, for instance, the sectioning of bacterial nanowires, which are produced by iron(III)-reducing bacteria as conductive appendages for electron transfer [108], to study their internal structure. Alternatively, nanoscale fabrication could be used to better understand biomineralization by bacteria in the environment. Milling experiments could also provide insight into the development and release of extracellular vesicles or viruses from cell membranes. In addition to microbiological applications, in general, subsurface imaging of any biological sample at a precisely defined location could become another application area, in particular, if porous features are to be expected.

Despite its power as an imaging device, the ability to obtain analytical information with HIM is still not widespread. However, an increasing number of instruments are being equipped with mass spectrometry (SIMS) detectors, which combine the high resolution provided by helium ions with elemental or isotope-selective quantification. This technology will remove one of the key differences between HIM and SEM. Other detectors, such as RBS and ionoluminescence, will further improve the capabilities of HIM to image and analyse biological samples. Advancements in the detection of transmitted helium ions with specifically designed ion detectors which are being developed as part of the npScope project mean that bio-imaging using HIM is likely to improve still further. Another important advancement that is anticipated to emerge in HIM instruments over the next few years is the ability to image samples at cryogenic temperature. This development would complement cryo-TEM and cryo-SEM, which are already established for studying hydrated and biological materials. Essentially, samples are rapidly frozen to cryogenic temperatures. This effectively fixes the samples without the requirement to use chemicals (e.g., glutaraldehyde), which can induce side reactions. The main technical requirement is a cryogenic sample stage to be mounted within the instrument, which can maintain the specimen temperature below −120 °C [109]. To the best of our knowledge, there is currently no HIM equipped with such a stage. However, the ability to combine cryogenic imaging of biological samples with the high-resolution and milling capabilities (see section “Nanofabrication”) would likely be of broad interest to the imaging community.

In this review paper, we have highlighted just some of the exciting developments which have been made with HIM in studies ranging from medical research to microbiology, plants, small animals, and geomicrobiology. From imaging white blood cells [2] to providing pseudo-3D micrographs of cartilage at unparalleled resolution [70], or from images of the nanopillar texture on the wings of a butterfly [12] to the precipitation of iron oxide minerals onto organic templates created by iron(II)-oxidizing bacteria [80], HIM bio-imaging offers enormous potential and we hope to see its adoption continuing to spread.

## References

[1] Ward B W, Notte J A, Economou N P (2006). J Vac Sci Technol, B: Microelectron Nanometer Struct–Process, Meas, Phenom.

[2] Ward B, Notte J A, Economou N P (2007). Photonics Spectra.

[3] Notte J, Ward B, Economou N, Hill R, Percival R, Farkas L, McVey S (2007). AIP Conf Proc.

[4] Scipioni L, Stern L A, Notte J, Sijbrandij S, Griffin B (2008). Adv Mater Processes.

[R5] 5Carl Zeiss Microscopy, White paper: Zeiss Orion Plus (2008), (not available online anymore).

[6] Joens M S, Huynh C, Kasuboski J M, Ferranti D, Sigal Y J, Zeitvogel F, Obst M, Burkhardt C J, Curran K P, Chalasani S H (2013). Sci Rep.

[7] Bell D C, Erdman N, Jepson M A, Rodenburg C, Inkson B J (2009). Microsc Microanal.

[8] Bell D (2011). Microsc Microanal.

[9] Bazou D, Behan G, Reid C, Boland J J, Zhang H Z J (2011). J Microsc (Oxford, U K).

[10] Bazou D, Santos-Martinez M J, Medina C, Radomski M W (2011). Br J Pharmacol.

[11] Arey B, Shutthanandan V, Orr G (2011). Microsc Microanal.

[12] Boden S A, Asadollahbaik A, Rutt H N, Bagnall D M (2012). Scanning.

[13] Boseman A, Nowlin K, Ashraf S, Yang J, LaJeunesse D (2013). Micron.

[14] Kim K-W (2012). Appl Microsc.

[15] Rice W L, Van Hoek A N, PÇunescu T G, Huynh C, Goetze B, Singh B, Scipioni L, Stern L A, Brown D (2013). PLoS One.

[16] Schürmann M, Frese N, Beyer A, Heimann P, Widera D, Mönkemöller V, Huser T, Kaltschmidt B, Kaltschmidt C, Gölzhäuser A (2015). Small.

[17] Leppänen M, Sundberg L-R, Laanto E, de Freitas Almeida G M, Papponen P, Maasilta I J (2017). Adv Biosyst.

[18] Sharma A, Schmidt M, Kiesel B, Mahato N K, Cralle L, Singh Y, Richnow H H, Gilbert J A, Arnold W, Lal R (2018). Front Microbiol.

[19] Said N, Chatzinotas A, Schmidt M (2019). Adv Biosyst.

[20] Vinner G K, Rezaie-Yazdi Z, Leppanen M, Stapley A G F, Leaper M C, Malik D J (2019). Pharmaceuticals.

[21] Veligura V, Hlawacek G, van Gastel R, Zandvliet H J W, Poelsema B (2014). J Phys: Condens Matter.

[22] Veligura V, Hlawacek G, Jahn U, van Gastel R, Zandvliet H J W, Poelsema B (2014). J Appl Phys.

[23] Veligura V, Hlawacek G, van Gastel R, Zandvliet H J W, Poelsema B (2015). J Lumin.

[24] Franklin T M W (2012). Scanning Ionoluminescence Microscopy with a Helium Ion Microscope.

[25] Sato C, Sato M, Ogawa S (2018). Int J Mol Med.

[26] Dowsett D, Wirtz T, Vanhove N, Pillatsch L, Sijbrandij S, Notte J (2012). J Vac Sci Technol, B: Nanotechnol Microelectron: Mater, Process, Meas, Phenom.

[27] Wirtz T, Vanhove N, Pillatsch L, Dowsett D, Sijbrandij S, Notte J (2012). Appl Phys Lett.

[28] Wirtz T, Dowsett D, Philipp P, Hlawacek G, Gölzhäuser A (2016). SIMS on the Helium Ion Microscope: A Powerful Tool for High-Resolution High-Sensitivity Nano-Analytics. Helium Ion Microscopy.

[29] Dowsett D, Wirtz T (2017). Anal Chem (Washington, DC, U S).

[30] Eyhusen S Carl Zeiss Microscopy White paper: Multi-Modal Characterization with Secondary Ion Mass Spectrometry on ZEISS ORION NanoFab.

[31] Klingner N, Heller R, Hlawacek G, Facsko S, von Borany J, Wilhelm R A (2016). Patentschrift Ionenmikroskopievorrichtung.

[32] Klingner N (2016). Ionenstrahlanalytik im Helium-Ionen-Mikroskop.

[33] Klingner N, Heller R, Hlawacek G, von Borany J, Notte J, Huang J, Facsko S (2016). Ultramicroscopy.

[34] Heller R, Klingner N, Hlawacek G, Hlawacek G, Gölzhäuser A (2016). Backscattering Spectrometry in the Helium Ion Microscope: Imaging Elemental Compositions on the nm Scale. Helium Ion Microscopy.

[35] Klingner N, Heller R, Hlawacek G, Facsko S, von Borany J (2019). Ultramicroscopy.

[36] Lovric J, Audinot J-N, Wirtz T (2019). Microsc Microanal.

[37] Kim K W (2013). Appl Microsc.

[38] Gölzhäuser A, Hlawacek G, Hlawacek G, Gölzhäuser A (2016). HIM of Biological Samples. Helium Ion Microscopy.

[39] Ward B, Hlawacek G, Gölzhäuser A (2016). The ALIS Story. Helium Ion Microscopy.

[40] Leppänen M (2020). Infection under the ion beam : focused ion beams and antibacterial properties of biomaterials.

[41] Inai K, Ohya K, Ishitani T (2007). J Electron Microsc.

[42] Everhart T E, Thornley R F M (1960). J Sci Instrum.

[43] Carl Zeiss Microscopy White Paper (2016): Zeiss ORION NanoFab.

[44] Tominski C, Lösekann-Behrens T, Ruecker A, Hagemann N, Kleindienst S, Mueller C W, Höschen C, Kögel-Knabner I, Kappler A, Behrens S (2018). Appl Environ Microbiol.

[45] Meldrum F C, Mann S, Heywood B R, Frankel R B, Bazylinski D A (1993). Proc R Soc London, Ser B.

[46] Moreno-Osorio J H, Benettoni P, Schmidt M, Stryhanyuk H, Schmitt-Jansen M, Pinto G, Pollio A, Frunzo L, Lens P N L, Richnow H H (2019). FEMS Microbiol Ecol.

[47] Imlay J A (2006). Mol Microbiol.

[48] Rouault T A, Tong W-H (2005). Nat Rev Mol Cell Biol.

[49] Klingner N, Hlawacek G, Heller R, von Borany J, Facsko S (2016). Nanometer Scale Time of Flight Back Scattering Spectrometry in the Helium Ion Microscope. European Microscopy Congress 2016: Proceedings.

[50] Pallon J, Yang C, Utui R J, Elfman M, Malmqvist K G, Kristiansson P, Sjöland K A (1997). Nucl Instrum Methods Phys Res, Sect B.

[51] Hell S W, Wichmann J (1994). Opt Lett.

[52] Mi Z (2015). Development and biological applications of high-resolution ion beam induced fluorescense microscopy.

[53] Breese M B H, Landsberg J P, King P J C, Grime G W, Watt F (1992). Nucl Instrum Methods Phys Res, Sect B.

[54] Marshall M M, Yang J, Hall A R (2012). Scanning.

[55] Hall A R (2013). Microsc Microanal.

[56] Woehl T J, White R M, Keller R R (2016). Microsc Microanal.

[57] Ziegler J F (2020). SRIM 2013 - The Stopping and Range of Ions in Matter.

[58] Mousley M, Eswara S, De Castro O, Bouton O, Klingner N, Koch C T, Hlawacek G, Wirtz T (2019). Beilstein J Nanotechnol.

[59] (2020). npSCOPE.

[60] Zeitvogel F, Burkhardt C J, Schroeppel B, Schmid G, Ingino P, Obst M (2017). Geomicrobiol J.

[61] Loftus A F, Joens M S, Dunn S E, Adams M W, Huynh C, Goetze B, Fitzpatrick J A J (2015). Microsc Microanal.

[62] Goetze B, Huynh C, Stern L, Wu H, Ferranti D, Ananth M (2013). Microsc Microanal.

[63] Uryu K, Soplop N, Acehan D, Winer B Y, Fischetti V A, Sheahan T, Rice C M, Hsu M, Robbiani M, Santulli G (2016). Microsc Microanal.

[64] Uryu K, Rice C M, Catanese M T, Santulli G, Totary-Jain H, Huynh C, Goetze B (2017). Microsc Microanal.

[65] P unescu T G, Shum W W C, Huynh C, Lechner L, Goetze B, Brown D, Breton S (2014). Mol Hum Reprod.

[66] Laufer K, Niemeyer A, Nikeleit V, Halama M, Byrne J M, Kappler A (2017). FEMS Microbiol Ecol.

[67] Bidlack F B, Huynh C, Marshman J, Goetze B (2014). Front Physiol.

[68] Tsuda T, Nemoto N, Kawakami K, Mochizuki E, Kishida S, Tajiri T, Kushibiki T, Kuwabata S (2011). ChemBioChem.

[69] Golding C G, Lamboo L L, Beniac D R, Booth T F (2016). Sci Rep.

[70] Vanden Berg-Foels W S, Scipioni L, Huynh C, Wen X J (2012). J Microsc (Oxford, U K).

[71] Huyuan C, Marshman J, Dobeck J, Goetze B, Bidlack F B (2013). Microsc Microanal.

[72] Paunescu T G, Breton S, Brown D (2014). Physiol News.

[73] Tsuji K, Păunescu T G, Suleiman H, Xie D, Mamuya F A, Miner J H, Lu H A J (2017). Sci Rep.

[74] Herrera M G, Pizzuto M, Lonez C, Rott K, Hütten A, Sewald N, Ruysschaert J-M, Dodero V I (2018). Nanomedicine (N Y, NY, U S).

[75] Curtin A E, Chiaramonti A N, Sanders A W, Ciesielski P N, Chapple C, Mosier N, Donohoe B S (2014). Microsc Microanal.

[76] Bandara C D, Singh S, Afara I O, Wolff A, Tesfamichael T, Ostrikov K, Oloyede A (2017). ACS Appl Mater Interfaces.

[77] Bandara C C D I M (2017). Characterisation of the bactericidal efficiancy of natural nanotopography using dragonfly.

[78] Shahali H, Hasan J, Mathews A, Wang H, Yan C, Tesfamichael T, Yarlagadda P K D V (2019). J Mater Chem B.

[79] Belianinov A, Halsted M C, Burch M J, Songkil K, Retterer S T (2017). Microsc Microanal.

[80] Byrne J M, Schmidt M, Gauger T, Bryce C, Kappler A (2018). Environ Sci Technol Lett.

[81] Almeida G M F, Leppänen M, Maasilta I J, Sundberg L-R (2018). Res Microbiol.

[82] Prasad K, Recek N, Zhou R, Zhou R, Aramesh M, Wolff A, Speight R E, Mozetič M, Bazaka K, Ostrikov K (2019). Sustainable Mater Technol.

[83] de Souza W, Attias M (2015). J Struct Biol.

[84] Gadelha A P R, Benchimol M, de Souza W (2015). J Struct Biol.

[85] de Souza W, Attias M (2018). Exp Parasitol.

[86] Starr M P, Seidler R J (1971). Annu Rev Microbiol.

[87] Sockett R E (2009). Annu Rev Microbiol.

[88] (2020). WHO priority pathogens list for Research and Development of new antibiotics.

[89] LeTourneau M K, Marshall M M, Thomashow L S, Harsh J B (2015). Microsc Microanal.

[90] Kniggendorf A-K, Gaul T W, Meinhardt-Wollweber M (2011). Microsc Res Tech.

[91] Samuels T, Bryce C, Landenmark H, Marie‐Loudon C, Nicholson N, Stevens A H, Cockell C (2020). Microbial Weathering of Minerals and Rocks in Natural Environments. Biogeochemical Cycles.

[92] Bryce C, Franz-Wachtel M, Nalpas N C, Miot J, Benzerara K, Byrne J M, Kleindienst S, Macek B, Kappler A (2018). Appl Environ Microbiol.

[93] Melton E D, Swanner E D, Behrens S, Schmidt C, Kappler A (2014). Nat Rev Microbiol.

[94] Nordhoff M, Tominski C, Halama M, Byrne J M, Obst M, Kleindienst S, Behrens S, Kappler A (2017). Appl Environ Microbiol.

[95] Dohnalkova A, Arey B, Varga T, Miller M, Kovarik L (2017). Microsc Microanal.

[96] Lybrand R A, Austin J C, Fedenko J, Gallery R E, Rooney E, Schroeder P A, Zaharescu D G, Qafoku O (2019). Sci Rep.

[97] Qafoku O, Lybrand R A, Shutthanandan V, Gallery R E, Austin J C, Schroeder P A, Fedenko J, Rooney E, Zaharescu D G (2019). Microsc Microanal.

[98] Rasa K, Heikkinen J, Hannula M, Arstila K, Kulju S, Hyväluoma J (2018). Biomass Bioenergy.

[99] LeTourneau M K, Marshall M J, Cliff J B, Bonsall R F, Dohnalkova A C, Mavrodi D V, Devi S I, Mavrodi O V, Harsh J B, Weller D M (2018). Environ Microbiol.

[100] Chen S-C, Musat N, Lechtenfeld O J, Paschke H, Schmidt M, Said N, Popp D, Calabrese F, Stryhanyuk H, Jaekel U (2019). Nature.

[101] Hill R, Notte J A, Scipioni L (2012). Scanning Helium Ion Microscopy. Advances in Imaging and Electron Physics.

[102] Shorubalko I, Pillatsch L, Utke I, Hlawacek G, Gölzhäuser A (2016). Direct–Write Milling and Deposition with Noble Gases. Helium Ion Microscopy.

[103] Vinner G K, Richards K, Leppänen M, Sagona A P, Malik D J (2019). Pharmaceutics.

[104] Bandara C D, Ballerin G, Leppänen M, Tesfamichael T, Ostrikov K K, Whitchurch C B (2020). ACS Biomater Sci Eng.

[105] Decelle J, Veronesi G, Gallet B, Stryhanyuk H, Benettoni P, Schmidt M, Tucoulou R, Passarelli M, Bohic S, Clode P (2020). Trends Cell Biol.

[106] Chen X, Udalagama C N B, Chen C-B, Bettiol A A, Pickard D S, Venkatesan T, Watt F (2011). Biophys J.

[107] Sanders A W, Jeerage K M, Schwartz C L, Curtin A E, Chiaramonti A N (2014). Microsc Microanal.

[108] Reguera G, McCarthy K D, Mehta T, Nicoll J S, Tuominen M T, Lovley D R (2005). Nature.

[109] Stokes D J, Mugnier J-Y, Clarke C J (2004). J Microsc (Oxford, U K).

